# Programmable microbial ink for 3D printing of living materials produced from genetically engineered protein nanofibers

**DOI:** 10.1038/s41467-021-26791-x

**Published:** 2021-11-23

**Authors:** Anna M. Duraj-Thatte, Avinash Manjula-Basavanna, Jarod Rutledge, Jing Xia, Shabir Hassan, Arjirios Sourlis, Andrés G. Rubio, Ami Lesha, Michael Zenkl, Anton Kan, David A. Weitz, Yu Shrike Zhang, Neel S. Joshi

**Affiliations:** 1 John A. Paulson School of Engineering and Applied Sciences, Harvard University, Cambridge, MA USA; 2 Wyss Institute for Biologically Inspired Engineering, Harvard University, Boston, MA USA; 3 Department of Chemistry and Chemical Biology, Northeastern University, Boston, MA USA; 4 Division of Engineering in Medicine, Department of Medicine, Brigham and Women’s Hospital, Harvard Medical School, Cambridge, MA USA; 5 Present Address: Department of Biological Systems Engineering, Virginia Polytechnic Institute and State University, Blacksburg, VA USA

**Keywords:** Synthetic biology, Biomaterials

## Abstract

Living cells have the capability to synthesize molecular components and precisely assemble them from the nanoscale to build macroscopic living functional architectures under ambient conditions. The emerging field of living materials has leveraged microbial engineering to produce materials for various applications but building 3D structures in arbitrary patterns and shapes has been a major challenge. Here we set out to develop a bioink, termed as “microbial ink” that is produced entirely from genetically engineered microbial cells, programmed to perform a bottom-up, hierarchical self-assembly of protein monomers into nanofibers, and further into nanofiber networks that comprise extrudable hydrogels. We further demonstrate the 3D printing of functional living materials by embedding programmed *Escherichia coli* (*E. coli*) cells and nanofibers into microbial ink, which can sequester toxic moieties, release biologics, and regulate its own cell growth through the chemical induction of rationally designed genetic circuits. In this work, we present the advanced capabilities of nanobiotechnology and living materials technology to 3D-print functional living architectures.

## Abstract (web summary)

Living cells can precisely assemble to build 3D functional architectures. Here the authors produce an extrudable microbial ink entirely from the engineered cells, which can be further programmed to 3D print functional living materials.

## Introduction

3D bioprinting technology, which is relatively well-established for printing mammalian cells in the context of tissue engineering, has more recently been applied to print microbial cells for biotechnological and biomedical applications^1–8^. Although inkjet printing, contact printing, screen printing, and lithographic techniques have been explored to print microbes, extrusion-based bioprinting has become one of the most widely used techniques due to its simplicity, compatibility with a variety of bioinks, and cost-effective instrumentation^2,9–11^. In an early example of this concept, a mixture of alginate and *E. coli* was extruded onto a printing surface consisting of calcium chloride, upon which the alginate molecules crosslink to form a solidified gel^7^. A similar ionic crosslinking strategy was exploited to generate photocurrent with 3D printed cyanobacteria^12^. In another approach, a multi-material bioink comprised of hyaluronic acid, *κ*-carrageenan, fumed silica, and a photo-initiator was employed to 3D-print *Pseudomonas putida* and *Acetobacter xylinum*. Also, photo-crosslinked pluronic F127 acrylate-based bioinks have been utilized to print living, responsive materials/devices, and catalytically active living materials^4,6,13^.

An alternative strategy made use of freeze-dried *Saccharomyces cerevisiae* as the primary component of a bioink formulation consisting of nanocellulose, polyethylene glycol dimethacrylate, and a photoinitiator^3^. The latter approach yielded remarkably high cell densities of 10^9^ cells ml^−1^, but the need for freeze-drying could significantly affect the survival rate of other microbial species as well as their thixotropic behavior. In an interesting approach, the viscoelastic gel-like characteristics of *Bacillus subtilis* (*B. subtilis*) biofilms facilitated direct printing. However, the wild-type biofilms were unable to maintain the printed line widths (as they expanded three-fold in width after printing), while the engineered variants had lower storage modulus and viscosity that restricted their printing in multiple layers^8^. In yet another strategy, a fused deposition modeling was adapted to deposit molten agarose (75 °C) containing *B. subtilis* spores onto a cold substrate (16 °C), resulting in hardened patterns upon cooling^5^. Here, the high-temperature processing works well for spores, but limits applicability to a wide range of cell types.

Although the above examples demonstrate that many bioink designs have already been explored, none so far have fully leveraged the genetic programmability of microbes to rationally control the mechanical properties of the bioink. This would be advantageous for several reasons, including the possibilities of more sustainable manufacturing practices, raw material fabrication in resource-poor environments (terrestrial or extra-terrestrial), and enhanced material performance through bio-inspired design and the precision of genetic programming. In contrast to the examples described above, we envisioned to (1) design an extrudable bioink that had high print fidelity, (2) produce the bioink entirely from engineered microbes by a bottom-up approach and (3) create a programmable platform that would enable advanced functions for the macroscopic 3D living architectures, and thereby push the emerging field of living materials to unexplored frontiers^3–10,12–28^.

In this work, we present microbial ink that is produced entirely from the genetically engineered *E. coli* biofilms. We show the detailed characterization of the microbial ink and demonstrate its structural and shape integrity. Further, by embedding genetically programmed *E. coli* cells in the microbial ink, we demonstrate the 3D printing of therapeutic living material, sequestration living material, and regulatable living material.

## Results

### Design strategy and production of microbial ink

**Fig. 1:**
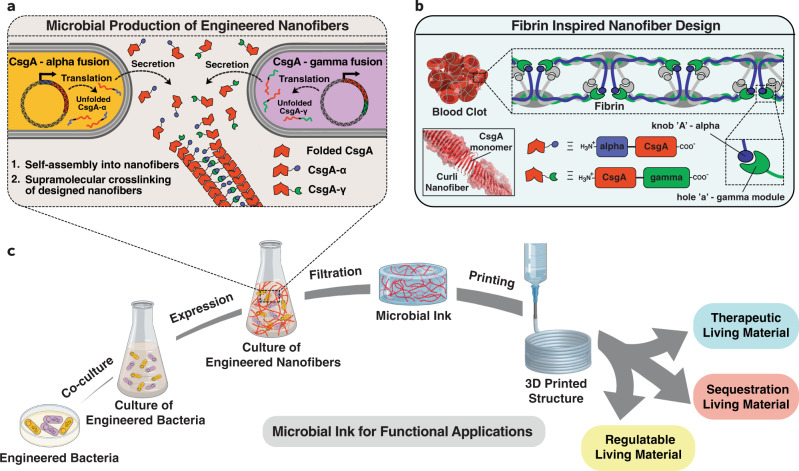
Schematics of the design strategy, production, and functional applications of microbial ink. **a** *E. coli* was genetically engineered to produce microbial ink by fusing *α* (knob) and *γ* (hole) protein domains, derived from fibrin to the main structural component of curli nanofibers, CsgA. Upon secretion, the CsgA-*α* and CsgA-*γ* monomers self-assemble into nanofibers crosslinked by the knob-hole binding interaction. **b** The knob and hole domains are derived from fibrin, where they play a key role in supramolecular polymerization during blood clot formation. **c** The protocol to produce microbial ink from the engineered protein nanofibers involves standard bacterial culture, limited processing steps, and no addition of exogenous polymers. Microbial ink was 3D printed to obtain functional living materials.

There is a critical need to develop advanced bioinks with tunable mechanical strength, high cell viability, and high print fidelity^11^. A printable bioink requires a viscosity low enough to facilitate extrusion, but high enough to retain its shape after printing^2^. In this regard, shear-thinning hydrogels, which decrease their viscosity with increasing shear stress, are an attractive option. Moreover, it should be noted that bioinks are biocompatible materials typically meant to recapitulate an extracellular matrix (ECM) to provide a congenial environment for the growth of living cells with predefined structures and functions. We envisioned that instead of embedding microbes in an ECM-mimicking bioink, we could repurpose the ECM of the microbial biofilm itself to serve as a programmable bioink. This idea is built on our earlier work, wherein we showed that the native proteinaceous curli nanofibers of an *E. coli* biofilm ECM can be genetically engineered by fusing functional peptides/proteins to the curli CsgA monomer to produce a shear-thinning hydrogel^18^. However, in order to create a bioink with the desired viscoelastic performance, we introduced a genetically programmed crosslinking strategy, inspired by fibrin (Fig. 1)^29^. Fibrin is a protein involved in the clotting cascade, which activates its polymerization to form blood clots. Fibrin’s polymerization is driven in part by noncovalent interactions between an alpha-chain domain present on the N-terminus of one fibrin monomer (i.e., the “knob” domain) and a gamma-chain domain on the C-terminus (i.e., the “hole” domain) of an adjacent monomer^29^. Our microbial ink design repurposes this binding interaction between alpha and gamma modules, i.e., the knob-hole interaction, to introduce non-covalent crosslinks between nanofibers and enhance mechanical robustness while maintaining shear-thinning properties (Fig. 1). It should also be noted that fibers formed from the self-assembly of CsgA are highly stable and resistant to proteolytic, detergent-induced, and heat-based denaturation^30^.

**Fig. 2:**
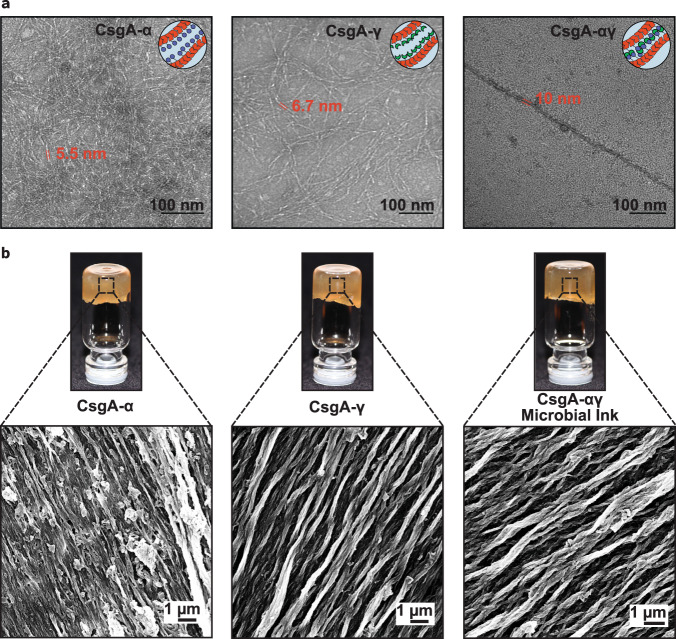
Optical and electron microscopy images of functional curli nanofibers and the corresponding hydrogels. **a** Transmission electron microscope (TEM) images of self-assembled nanofibers of CsgA-*α*, CsgA-*γ* and CsgA-*αγ* (co-culture of CsgA-*α* and CsgA-*γ*) after recombinant expression. Representative images from three independent samples were reported. **b** Optical images of CsgA-*α*, CsgA-*γ* and microbial ink CsgA-*αγ* hydrogels with the corresponding field-emission scanning electron microscope (FESEM) images show the presence of aligned microscopic fiber bundles. Representative images from three independent samples were reported.

Using the Biofilm Integrated Nanofiber Display (BIND) technology developed in our laboratory^25^, we genetically grafted the “knob” and “hole” protein domains to the N- and C-terminus of CsgA, respectively, to create the fusion proteins CsgA-*α* and CsgA-*γ* (Fig. 1, Supplementary Table 1). CsgA-*α* and CsgA-*γ* were expressed separately in engineered *E. coli* strain PQN4 along with the auxiliary curli genes necessary for secretion and assembly, and the resulting curli nanofibers were imaged using transmission electron microscopy (TEM). After staining with 1% uranyl formate, the nanofibers showed diameters of ~5.5 nm (CsgA-*α*) and ~6.7 nm (CsgA-*γ*) (Fig. 2a). Notably, curli nanofibers composed of wild-type CsgA have diameters of ~4 nm^25^. Thus, the observed trend in nanofiber diameters is qualitatively consistent with the relative sizes of the fused domains – 11 amino acids for “knob” (CsgA-*α*) and 127 amino acids for “hole” (CsgA-*γ*). When the two types of *E. coli* cells, each expressing either CsgA-*α* or CsgA-*γ*, were co-cultured (CsgA-*αγ*), they produced nanofibers that display “knob” and “hole” domains. TEM imaging showed three nanofiber populations with diameters of ~5.5, ~6.7, and ~10 nm (Fig. 2a, Supplementary Fig. 1). We attribute the 10 nm diameter nanofibers to supramolecular crosslinking mediated by noncovalent interactions of the “knob” and “hole” domains (Fig. 1). We then created hydrogels from the microbial cultures using a simple filtration protocol, as described in our earlier reports^18^. Briefly, the microbial culture was filtered through a nylon membrane to concentrate the curli nanofibers, and then treated with guanidinium chloride, nuclease, and sodium dodecyl sulfate to obtain cell-free hydrogels composed of the designed curli nanofibers (Fig. 2b)^18^. Field-emission scanning electron microscopy (FESEM) indicated a fibrous microstructure for all three hydrogels (CsgA-*α*, CsgA-*γ* and the co-culture CsgA-*αγ*), with the fiber alignment suggesting hierarchical assembly through the lateral association of functional curli nanofibers (Fig. 2b).

### Characterization and 3D printing of microbial ink

**Fig. 3:**
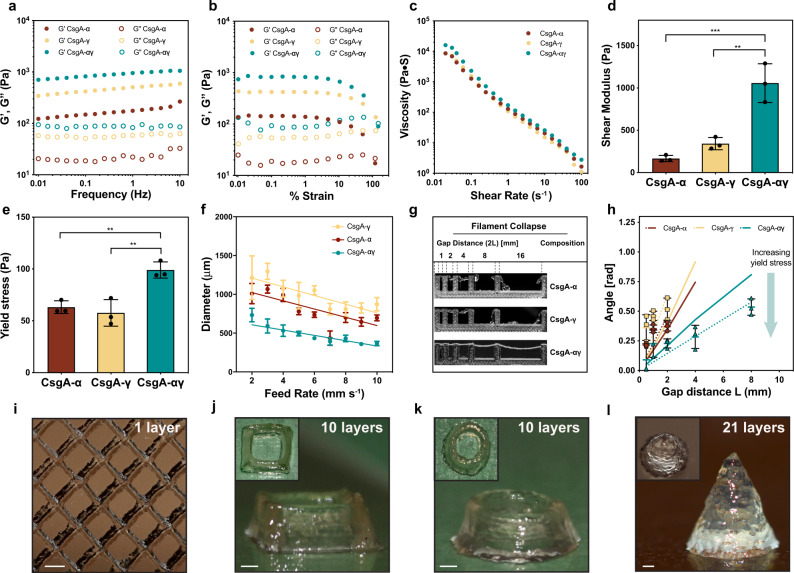
Rheological properties and 3D printing of CsgA-*α*, CsgA-*γ* and microbial ink CsgA-*αγ*. The storage modulus (G’) and loss modulus (G”) under frequency sweep (**a**) and oscillatory sweep (**b**). **c** Viscosity as a function of shear rate, **d** Shear modulus (*n* = 3) **p = 0.0014, ***p = 0.0004 and **e** yield stress (*n* = 3) **p = 0.0061, **p = 0.003, one-way ANOVA followed by Dunnett’s test. **f** Printed line diameter as a function of feed rates ranging from 2 to 10 mm s^−1^ at 20 psi pressure (*n* > 10). **g** Images of filament collapse test and **h**. the plot of deflection angle versus pillar gap distances (*n* = 3). Experimental data: solid line, theoretically predicted data: dotted line. Data represented as mean ± standard deviation. **p ≤ 0.01, ***p ≤ 0.001, one-way ANOVA followed by Dunnett’s test. 3D printed structures using the microbial ink CsgA-*αγ* **i** single-layer grid, **j** 10-layer square, **k** 10-layer circle, and **l** 21-layer solid cone. Insets in (**j**–**l**). are corresponding top views. Scale bar 1 mm.

We investigated the rheological properties of CsgA-*α*, CsgA-*γ,* and CsgA-*αγ* hydrogels, to validate their potential as extrudable bioinks. Frequency sweep experiments revealed that the storage modulus (G’) of the CsgA-*αγ* hydrogel was several-fold higher than that of the CsgA-*α* and CsgA-*γ* hydrogels alone, while the G’ of all the hydrogels were higher than their loss modulus (G”) by an order of magnitude (Fig. 3a). Strain sweep experiments showed that the hydrogels were stable up to ~10% strain, above which a crossover point is observed as G’ decreased and G” increased. (Fig. 3b). The viscosity of all the hydrogels was also found to decrease with increasing shear rate, which indicates their shear-thinning behavior (Fig. 3c). Similarly, the shear modulus (G) of CsgA-*αγ* was higher than that of CsgA-*α* and CsgA-*γ* by 6- and 3-fold, respectively (Fig. 3d). The yield stress (*σ*_*y*_) of CsgA-*αγ* was nearly twice that of CsgA-*α* and CsgA-*γ* (Fig. 3e). From all the above experiments, it is clear that supramolecular crosslinking of “knob” and “hole” domains in CsgA-αγ significantly increased the G’, G, *σ*_*y*,_ and viscosity, making it better suited than CsgA-*α* or CsgA-*γ* for extrusion printing^2,11^. On the other hand, when CsgA-*α* and CsgA-*γ* fibers expressed in separate cultures were mixed (CsgA-*αγ*-mix) in a 1:1 volume ratio also yielded hydrogels with rheological properties similar to CsgA-*αγ*, which further confirms our hypothesis about supramolecular crosslinking between the complementary fibers (Supplementary Figs. 2–3).

We then tested the printability of the CsgA-*α*, CsgA-*γ,* and CsgA-*αγ* hydrogel-based bioinks using a customized 3D printer (Supplementary Fig. 4). First, the hydrogel-based bioinks were extruded under a range of feed rates (2–10 mm s^−1^) and pressures (20–40 psi) to understand their printing performance (Fig. 3f, Supplementary Figs. 5−10). The printed line widths of CsgA-*α* and CsgA-*γ* bioinks were nearly two times that of CsgA-*αγ* for the same feed rate and pressure, indicating the superior structural integrity of CsgA-*αγ* (Fig. 3f, Supplementary Figs. 5–10). Subsequently, we tested the shape fidelity of the bioinks according to a published protocol (filament collapse test) to provide a quantitative comparison between bioinks^31^. A single line (filament) of each bioink was extruded at a nozzle moving speed of 5 mm s^−1^ on a platform with pillars at known gap distances, to bridge the pillar gaps (Fig. 3g, Supplementary Movie 1). The CsgA-*α* and CsgA-*γ* bioinks were unable to bridge gap distances of 8 mm and above, whereas the CsgA-*αγ* bioink was able to support its own weight for gap distances as large as 16 mm. This remarkable property, along with the higher *σ*_*y*_ and viscosity, suggests that the supramolecular knob-hole crosslinks allow for fast reassembly after extrusion—an important feature for optimal bioink performance^32,33^. Shape fidelity was assessed quantitatively by measuring the angles of deflection of the overhung bioink fibers under gravitational force (Fig. 3h)^31^. When the angles of deflection are plotted against the half gap distances, the resulting slope will decrease for the bioink with higher *σ*_*y*_^31^_._ This experimental data was also consistent with the reported theoretical model, while the deviation of experimental and predicted slopes was also in line with the original report, which observed that the model overestimated the angles of deflection, likely due to the exclusion of gel viscoelasticity and surface tension from the theoretical model^31^. We then utilized the CsgA-*αγ* bioink to 3D-print defined patterns and shapes. A single-layer grid shows the finer line structures of the printed pattern obtained with a resolution of ~300 μm from a 27 G needle (Fig. 3i). The multilayered architectures presented in Fig. 3j (10-layered square), Fig. 3k (10-layered circle) and Fig. 3l (21-layered solid cone) demonstrated the structural integrity of the microbial ink.

### 3D printing of functional living materials

**Fig. 4:**
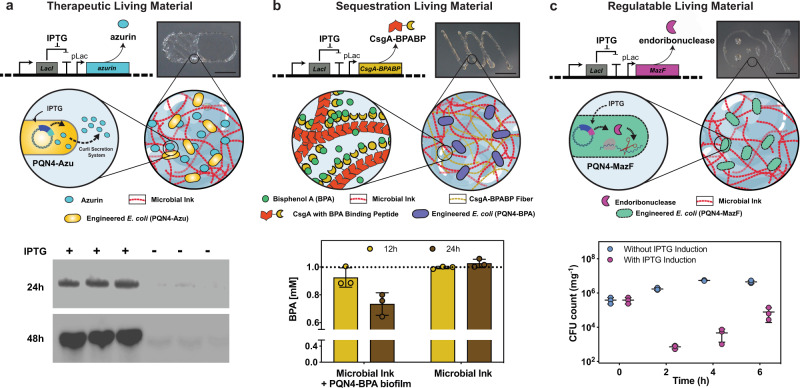
3D printing of functional living materials. **a** Genetic design of *E. coli* (PQN4-Azu) cells, programmed to secrete an anticancer biologic drug azurin along with image of printed living material (top), and the incorporation of PQN4-Azu cells into the CsgA-*αγ* microbial ink (middle). Western blot (bottom) shows the difference in azurin detected in the supernatant of the printed structure with and without IPTG induction. **b** Genetic design of *E. coli* (PQN4-BPA), programmed to produce extracellular fibers displaying BPA-binding peptide (CsgA-BPABP) along with image of printed living material (top), and incorporation of CsgA-BPABP biofilm into microbial ink (middle). After 12 and 24 h, the BPA concentration (bottom) in the supernatant of the printed structure was analyzed by LCMS. Dotted line represents the initial BPA concentration of 1 mM. *n* = 3. **c** Genetic design of *E. coli* (PQN4-MazF) cells, programmed to express an endoribonuclease MazF, that inhibits/arrests cell growth along with image of printed living material (top), and incorporation of PQN4-MazF cells into microbial ink (middle). CFU count from printed structure over time, with and without IPTG induction (bottom). *n* = 3. Scale bar 5 mm. Data represented as mean ±  standard deviation.

After demonstrating the printing performance of the microbial ink, we introduced the genetically engineered microbes to the hydrogel to produce 3D-printed living functional architectures. Herein, we present a living material for therapeutic applications, wherein a chemical inducer, isopropyl *β*-D-1-thiogalactopyranoside (IPTG) was utilized to signal the programmed *E. coli* (PQN4-Azu) to synthesize on demand an anticancer biologic drug, azurin, and secrete it into the extracellular milieu (Fig. 4a, Supplementary Fig. 11)^34^. The microbial ink, laden with PQN4-Azu cells, was used to print a 2D capsule pattern that was incubated in the lysogeny broth (LB) media with (+) and without (−) the inducer IPTG. After 24 and 48 h of incubation, the detection of the secreted azurin by an anti-azurin antibody illustrated the functioning of the 3D-printed therapeutic living material (Fig. 4a). Next, we produced a living material designed to sequester a toxic chemical, Bisphenol A (BPA). For this demonstration, we grafted a BPA-binding peptide domain to CsgA (CsgA-BPABP) and loaded the PQN4 cells expressing CsgA-BPABP into the microbial ink (Fig. 4b, Supplementary Fig. 12)^35^. After printing a 2D pattern with the cell-laden ink, the pattern was incubated in LB media with 1 mM BPA. Liquid chromatography-mass spectroscopy (LC-MS) analysis showed that the microbial ink embedded with CsgA-BPA biofilm sequestered nearly 8% and 27% of the BPA after 12 and 24 h of incubation, respectively, while a negative control pattern made with microbial ink only, showed no appreciable BPA sequestration (Fig. 4b). Finally, we show that the cell growth within the printed material can be regulated by inducing a genetic circuit (Fig. 4c, Supplementary Fig. 13). To accomplish this, *E. coli* (PQN4-MazF) cells were programmed to express (upon induction with IPTG) the endoribonuclease toxin, MazF, that inhibits protein synthesis by cleaving mRNA, and can arrest cell growth and/or lead to cell death^36^. PQN4-MazF cells in the printed structure were found to proliferate in the absence of IPTG, but after 2 h of IPTG induction, the colony-forming unit (CFU) count reduced by nearly two orders of magnitude due to the expression of MazF (Fig. 4c). However, subsequently, the cell growth was restored to some extent, likely due to the native MazFE toxin-antitoxin system in *E. coli* (Fig. 4c)^36^. Such a regulation system can be further engineered to effectively control the cell growth and/or to induce cell death depending on the need.

In summary, we have genetically engineered the ECM of *E. coli* biofilms to produce a shear-thinning hydrogel by supramolecular crosslinking of fibrin-inspired recombinant protein nanofibers. Instead of using an external biocompatible material as the bioink, we have shown that a cell-laden bioink with target rheological and functional properties can be created purely through genetic engineering and minimal processing. The printability, structural integrity, and print fidelity of the microbial ink was demonstrated with the aid of detailed rheological studies and filament collapse tests. By incorporating programmed microbes/biofilms into the microbial ink, we have 3D-printed living materials that can be chemically induced to release the anticancer drug azurin, remove BPA from their surroundings, and regulate their own cell growth. The microbial ink design can be further customized for various biotechnological and biomedical applications using the ever-growing toolkit of biological parts being developed by synthetic biologists. Especially if combined with other materials technologies, such as those that are already incorporating living cells into structural building materials^37^, our microbial bioink could also be particularly useful for structure building in space or extraterrestrial habitats, where raw material transport is difficult, making on-demand generation of building materials from very limited resources essential^37^.

## Methods

### Plasmids for production of microbial ink

The CsgA-*α* construct was designed by fusing the fibrin-derived alpha (knob) peptide to the N-terminus of CsgA with an intervening 12-amino-acid flexible linker. However, in case of CsgA-*γ* construct, the gene encoding the fibrin-derived gamma (hole) protein was fused to the C-terminus of CsgA with an intervening 36-amino-acid flexible linker. Both genes encoding CsgA-*α* and CsgA-*γ* were synthesized (Integrated DNA Technologies) and cloned into pET21d vector using isothermal Gibson assembly (New England Biolabs). Both pET21dCsgA-*α* and pET21dCsgA-*γ* plasmids were transformed into PQN4, an *E. coli* cell strain derived from LSR10 (MC4100, Δ*csgA*, λ(DE3), Cam^R^) with the deletion of curli operon (∆csgBACEFG). Both pET21dCsgA-*α* and pET21dCsgA-*γ* plasmids contained curli operon genes co-transcribed with the engineered csgA, i.e., *csgC*, *csgE*, *csgF*, and *csgG*, which encodes the proteins necessary for the biosynthesis of curli fibers. In both these plasmids, the *csgB* gene was deleted from the curli operon in order to secrete CsgA fused alpha or gamma proteins and self-assemble them to functional curli fibers (CsgA-*α* or CsgA-*γ*) in the culture medium, without anchoring (Δ*csgB*) to the bacterial surface.

### Plasmids used to make functional microbial ink

The pET21dAzu plasmid was created similar to that of pET21dCsgA-*α* by replacing the gene of csgA-*α* with that of azurin, while we retained the SEC (N-terminal signal sequence) and N22 (N-terminal curli-specific targeting sequence) to allow secretion of azurin into the extracellular milieu. In case of pET21dCsgA-BPA plasmid (similar to pET21dCsgA-*γ*), the gene encoding BPA binding peptide was fused to the C-terminus of CsgA via a 36-amino-acid flexible linker. The MazF plasmid pL-MazF was derived from IPTG inducible plasmid pL6FO, and contains LacI repressor and kanamycin resistance genes, with a pLac promoter upstream of a *mazF* sequence derived from the genome of *E. coli* K-12 MG1655 (EcoCyc Accession EG11249)^38^. All plasmids used for creating functional microbial inks were transformed into *E. coli* strain PQN4.

### Microbial production of engineered nanofibers

pET21dCsgA-*α* and pET21dCsgA-*γ* plasmids were transformed into PQN4 cells and streaked onto lysogeny broth (LB) agar plates containing 100 µg ml^−1^ carbenicillin and 0.5% glucose (mv^−1^) for catabolite repression of T7RNAP and incubated overnight at 37 °C. One colony was picked from each plate of PQN4CsgA-*α* and PQN4CsgA-*γ*, and cultured separately at 37 °C in 5 ml LB media, 100 µg ml^−1^ carbenicillin and 2% glucose (mv^−1^). The overnight cultures of PQN4CsgA-*α* and PQN4CsgA-*γ* were transferred to a fresh 500 ml LB media containing 100 µg ml^−1^ carbenicillin and cultured upon IPTG induction either separately or together. This mono- or co-culture of PQN4CsgA-*α* and PQN4CsgA-*γ* was placed in incubator shakers (225 rpm, 37 °C) for 48 h to express the engineered proteins CsgA-*α* and CsgA-*γ*, and self-assemble them into functional curli nanofibers.

### Preparation of CsgA-*α*, CsgA-*γ* and CsgA-*αγ* hydrogels (microbial ink)

The 48 h bacterial culture (500 ml) with CsgA-*α*, CsgA-*γ* or CsgA-*αγ* nanofibers was treated with 0.8 M (final concentration) guanidinium chloride (GdmCl) and stored at 4 °C for 1 h. Subsequently, the engineered nanofibers were concentrated on a polycarbonate membrane with 11 μm pores (EMD Millipore) using vacuum filtration. The engineered nanofibers deposited on filter membrane were treated with 8 M GdmCl for 5 min. After which, it was vacuum filtered and washed with sterile deionized (DI) water (50 ml) twice to remove the lysed bacterial debris. Then, the nucleic acids bound to the curli fibers were removed by 10 min incubation with nuclease solution (Benzonase, Sigma-Aldrich, 1.5 U ml^−1^), which was followed by water washes (50 ml twice). Finally, the engineered curli nanofibers present on the filter membrane were incubated with 5% (mv^−1^ in water) sodium dodecyl sulfate (gelator) for 5 min, followed by vacuum filtration and DI water washes (50 ml twice). The hydrogel of functional curli nanofibers (CsgA-*α*, CsgA-*γ* or CsgA-*αγ*) formed on the filter membrane was scraped off and stored at 4 °C.

### Field-emission scanning electron microscopy (FESEM) sample preparation and imaging

FESEM samples were prepared by fixing with 2% (w v^−1^) glutaraldehyde and 2% (w v^−1^) paraformaldehyde at room temperature, overnight. The samples were gently washed with water, and the solvent was gradually exchanged to ethanol with an increasing ethanol 15-min incubation step gradient (25, 50, 75, and 100% (v v^−1^) ethanol). The samples were then dried in a critical point dryer, placed onto SEM sample holders using silver adhesive (Electron Microscopy Sciences), and sputtered until they were coated in a 10–20 nm layer of Pt/Pd. Images were acquired using a Zeiss Ultra55 FESEM equipped with a field emission gun operating at 5–10 kV. Representative images from three independent samples were reported.

### TEM sample preparation and imaging

Ten microliters of the cultures of CsgA-*α*, CsgA-*γ* or CsgA-*αγ* was drop-casted onto formvar-carbon grids (Electron Microscopy Sciences), washed with DI water (thrice), and stained with 1% uranyl formate before analysis on a JEOL 1200 TEM. Representative images from three independent samples were reported.

### Optical Images

Optical images were acquired using a Canon EOS Rebel SL3 Digital SLR Camera equipped with XIT 58 mm 0.43 Wide Angle Lens and XIT 58 mm 2.2x Telephoto Lens. Representative images from three independent samples were reported.

### Rheology studies of the hydrogels

The viscoelastic properties of the hydrogels were determined using a Discovery Hybrid Rheometer-3 and TRIOS software (TA Instruments, New Castle, DE). Hydrogels were loaded between a Peltier plate and a 20 mm plate geometry. The excess sample was trimmed along the edge of the 20 mm plate. Samples were surrounded with mineral oil to prevent dehydration. Time sweep experiments were conducted under continuous oscillations at 0.1 Hz with an imposed shear strain of 0.5%. After the storage modulus (G’) reached a plateau, a frequency sweep routine was applied under an oscillatory shear strain of 0.5% with the frequency increasing from 0.01 to 10 Hz. The storage modulus (G’) and loss modulus (G”) were recorded for both time and frequency sweep. The plateau storage modulus in the time sweep experiment was taken as the linear shear modulus. To probe the strain dependence of viscoelastic properties, samples were subjected to a strain sweep routine at a frequency of 0.1 Hz with the oscillatory strain increasing from 0.01 to 100%. To measure the viscosity of samples, samples were loaded between a Peltier plate and a 20 mm plate and a flow sweep routine was performed with the shear rate increasing from 0.01 to 100 Hz. To determine the yield stress of samples, samples were subjected to a stress sweep routine at a frequency of 1 Hz with the oscillatory stress increasing from 1 to 200 Pa. The yield stress is defined as the oscillatory stress where the storage modulus decreases to 50% smaller than the linear shear modulus. Data obtained from at least three independent samples were reported.

### 3D Printing of the hydrogels (microbial ink)

Prior to printing, the bioinks were transferred into a 10-ml Luer-Lok™ syringe and centrifuged at 112 × *g* for 2 min to remove any air bubbles. The standard needle used was a 27 G tip with a premade ¼” blunt end from Fisnar. Bioprinting was performed using an ANET A8 (Shenzhen Anet Technology Co) 3D printer that was upgraded into an extrusion bioprinter. The bioprinting was first conducted at a range of feed rates (2–10 mm s^−1^) and pressures (20–40 psi) to understand their effects on the extrusion of the bioinks. For bioprinting of actual patterns, the feed rate was kept consistent at 2.5 mm s^−1^, with a constant pressure of ~20 psi. The patterns were designed using Solidworks 3D design software (Dassault Systèmes SE). The 3D STL files were sliced using the slic3r engine from Repetier Host, which served as the main program for operating the extrusion bioprinter^39^.

### Print fidelity test for the hydrogels (microbial ink)

Fidelity test namely filament collapse test was performed according to the previously published protocol^31^. A small structure with pillars featuring a series of spacing’s (2 L) was first 3D-printed. A single line (filament; green) of the bioink was extruded by the bioprinter from one side of the structure to the other end at a moderate nozzle moving speed of 5 mm s^−1^, suspending the bioink between the pillars (purple) and bridging the gaps. Photographs were taken from the side of the structure post-bioprinting to measure the structural integrity under gravitational force (F_g_), in the form of angles (*θ*) of the overhung bioink fibers (Supplementary Fig. 14). Fidelity data fitting and theoretical modeling were conducted also in accordance with the previous report^31^. Data obtained from at least three independent samples were reported.

### Preparation of functional microbial ink

The PQN4-Azu and PQN4-MazF cells were grown to OD 1 at 37 °C in 10 ml LB media, with 100 µg ml^−1^ carbenicillin and 50 µg ml^−1^ kanamycin, respectively. The 1 ml of microbial ink (CsgA-*αγ*) was evenly spread on the filter membrane and was incubated with 10 ml of bacterial culture (PQN4-Azu or PQN4-MazF) for 30 min to disperse the bacteria on the microbial ink and then vacuum filtered. In case of PQN4-BPA, the bacterial cells first were grown to OD 1 at 37 °C in 10 ml LB media with 100 µg ml^−1^ carbenicillin and 2% glucose (m v^−1^). Next, the cells were transferred to fresh LB media containing 100 µg ml^−1^ carbenicillin and the CsgA-BPA fibers were expressed by induction with 0.1 mM IPTG. After 24 h of expression, the engineered PQN4-BPA biofilm was incorporated to 1 ml of microbial ink (CsgA-*αγ*), as described above. The as-prepared microbial ink embedded with programmed bacterial cells/biofilms were utilized for 3D printing.

### Detection of secreted azurin

The 3D printed structures of microbial ink (CsgA-*αγ*) embedded with PQN4-Azu cells were immersed in LB media containing 100 µg ml^−1^ carbenicillin with or without 0.1 mM IPTG and incubated at 37 °C for 48 h. After 24 and 48 h of incubation, 0.5 ml of the culture media was collected for detection of azurin secreted by the microbial ink embedded with PQN4-Azu cells. The culture was concentrated on Amicon® Ultra-0.5 centrifugal filter (MiliporeSigma) to 20 µl. The protein present in concentrated supernatant were separated by NuPAGE 4–20% Tris-Glycine gels (ThermoFisher Scientific) and transferred to a nitrocellulose membrane using iBlot™ 2 Gel Transfer Device (ThermoFisher Scientific). The membrane was incubated with Goat Polyclonal Anti-Azurin Antibody (Origene CAT#: AB0048-100, Dilution 1:1000) at 4 °C overnight. After washing, the membranes were incubated with a secondary antibody—rabbit anti-goat IgG-HRP (abcam6741, Dilution 1:1000) for 1 h at room temperature. Chemiluminescence was detected using a FluorChem M system (ProteinSimple). Data obtained from at least three independent samples were reported.

### BPA binding analysis

The 3D printed structures of microbial ink (CsgA-*αγ*) embedded with PQN4-BPA biofilm were immersed in water with or without 1 mM BPA and incubated for 24 h. After 12 and 24 h the water spiked with BPA was collected to test BPA binding by the microbial ink embedded with PQN4-BPA biofilm. The presence of BPA was detected using Agilent 6460 Triple Quad LC/MS and Agilent 1290 Infinity HPLC by Small Molecule Mass Spectrometry Facility at Harvard University, MA, USA. Data obtained from at least three independent samples were reported.

### Cell growth under MazF

The 3D printed structures of microbial ink (CsgA-*αγ*) embedded with PQN4-MazF cells were immersed with LB media consisting of 100 µg ml^−1^ carbenicillin, with or without 0.1 mM IPTG at 37 °C for 6 h. The growth of PQN4-MazF cells in the microbial ink was monitored by collecting ~200 mg of the ink after 0, 2, 4, and 6 h. The collected microbial inks were transferred to the Eppendorf tubes and 1 ml of LB media was added to resuspend the ink, which was serially diluted, and plated on carbenicillin selective plates to obtain their CFU counts. Data obtained from at least three independent samples were reported.

### Statistics and reproducibility

All experiments presented in this Article were repeated at least three times (*n* ≥ 3) on distinct samples, as clearly specified in the figure legends or the relevant Methods sections. In all cases, data are presented as the mean and standard deviation. GraphPad Prism 8 software was used for plotting and analyzing data. For micrographs and optical images, we present representative images.

### Reporting summary

Further information on research design is available in the Nature Research Reporting Summary linked to this article.

## Supplementary information

Supplementary Information

Supplementary Video 1

Description of Additional Supplementary Files

Reporting Summary

## Data Availability

All relevant data supporting the findings of this study are available within the Article and its Supplementary Information or from the corresponding authors upon request. Source data are provided with this paper.

## Source data

Source Data
Peer Review File
