# Supplementary material for: Programmable microbial ink for 3D printing of living materials produced from genetically engineered protein nanofibers
Source: Nat Commun. 2021 Nov 23;12:6600. doi: 10.1038/s41467-021-26791-x (PMC8611031; doi:10.1038/s41467-021-26791-x)
Supplement: Supplementary file 1 — Supplementary Information [file 41467_2021_26791_MOESM1_ESM.pdf]

Supplementary Materials

Programmable Microbial Ink for 3D Printing of Living Materials Produced from Genetically Engineered Protein Nanofibers

Anna M. Duraj-Thatte[1,2,3,5,6,*], Avinash Manjula-Basavanna[2,3,5,*], Jarod Rutledge[2], Jing Xia[1], Shabir Hassan[4], Arjirios Sourlis[2], Andrés G. Rubio[4], Ami Lesha[4], Michael Zenkl[4], Anton Kan[2], David A. Weitz[1], Yu Shrike Zhang[4], Neel S. Joshi[1,2,3,*]

[1] John A. Paulson School of Engineering and Applied Sciences, Harvard University, Cambridge, Massachusetts, United States.

[2] Wyss Institute for Biologically Inspired Engineering, Harvard University, Boston, Massachusetts, United States.

[3] Department of Chemistry and Chemical Biology, Northeastern University, Boston, Massachusetts, United States.

[4] Division of Engineering in Medicine, Department of Medicine, Brigham and Women's Hospital, Harvard Medical School, Cambridge, Massachusetts, United States.

[5] These authors contributed equally to this work

[6] Present Address – Department of Biological Systems Engineering, Virginia Polytechnic Institute and State University, Blacksburg, Virginia, United States.

*Corresponding author

aduraj@vt.edu

avinash.manjulabasavanna@wyss.harvard.edu

ne.joshi@northeastern.edu

This file includes:

Supplementary Figures 1-14
Supplementary Table 1

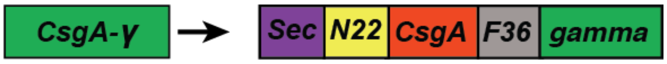

Name	Amino acid sequence
Sec	MKLLKVAAIAAIVFSGSALA
N22	GVVPQYGGGGNHGGGGNNSGPN
CsgA	GVVPQYGGGGNHGGGGNNSGPNSELNIYQYGGGNSALALQTDAR<br>NSDLTITQHGGGNGADVGQGSDDSSIDLTQRGFGNSATLDQWNGK<br>NSEMTVKQFGGGNGAAVDQTASNSSVNVTQVGFGNNATAHQY
Linker F36	GGSGSSGSGGSGGGSGSSGSGGSGGGSGSSGSGGSG
gamma	DAGDAFDGFDFGDDPSDKFFTSHNGMQFSTWDNDNDKFEGNCAE<br>QDGSGWWMNKCHAGHLNGVYYQGGTYSKASTPNGYDNGIIWAT<br>WKTRWYSMKKTTMKIIPFNRLTIGEGQQHHLGGAKQAGDV

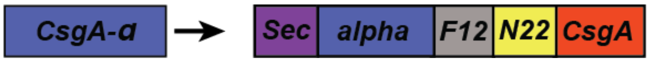

Name	Amino acid sequence
Sec	MKLLKVAAIAAIVFSGSALA
alpha	GPRVVERHQSA
Linker F12	GSGGSGGSGGSG
N22	GVVPQYGGGGNHGGGGNNSGPN
CsgA	GVVPQYGGGGNHGGGGNNSGPNSELNIYQYGGGNSALALQTDAR<br>NSDLTITQHGGGNGADVGQGSDDSSIDLTQRGFGNSATLDQWNGK<br>NSEMTVKQFGGGNGAAVDQTASNSSVNVTQVGFGNNATAHQY

Supplementary Table 1. Amino acid sequences of engineered curli nanofibers of CsgA-α and CsgA-γ that produce the microbial ink CsgA-$\alpha\gamma$.

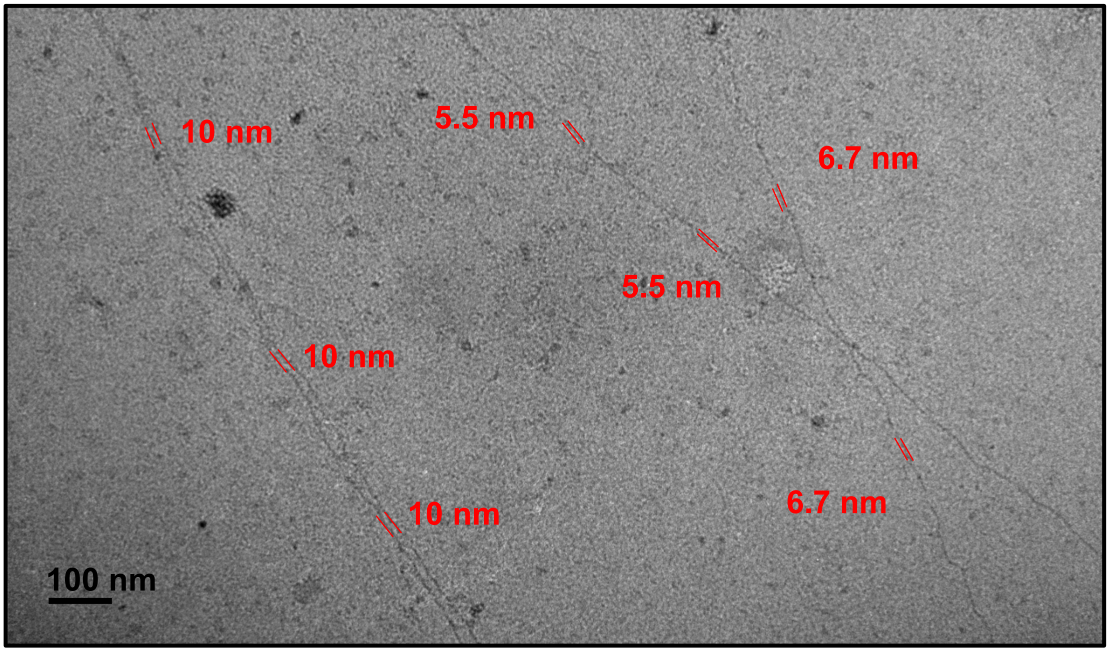

Supplementary Figure 1. Transmission electron microscope (TEM) image of a culture of CsgA-$\alpha\gamma$, showing the diameters of the nanofibers. The 10 nm fiber could be attributed to CsgA-$\alpha\gamma$ formed by the supramolecular crosslinking of alpha (knob) and gamma (hole) modules of CsgA-α and CsgA-γ, respectively. The nanofibers with 5.5 and 6.7 nm corresponds to CsgA-α and CsgA-γ, respectively. Representative images from three independent samples were reported.

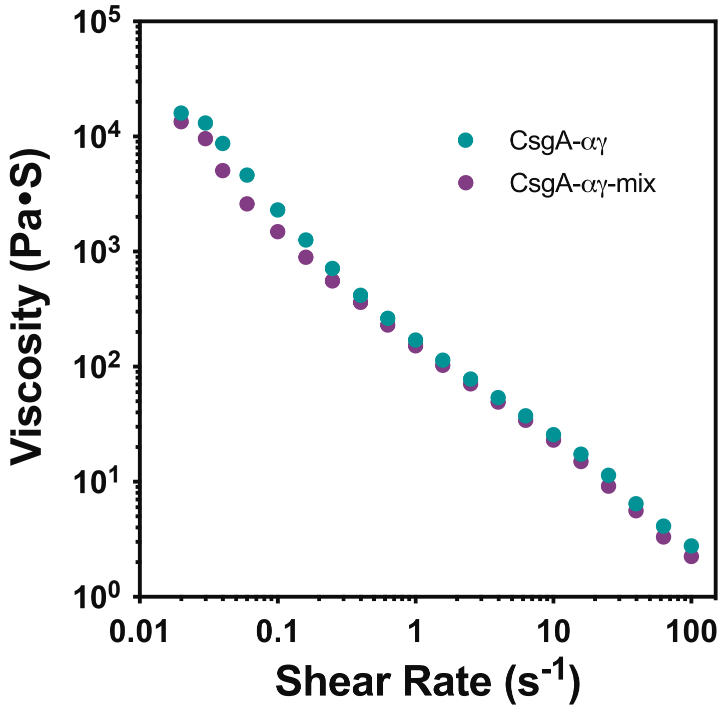

Supplementary Figure 2. Shear-thinning behavior of CsgA-$\alpha\gamma$ and CsgA-$\alpha\gamma$-mix. The decreasing viscosity with increasing shear rate indicates the shear-thinning property of CsgA-$\alpha\gamma$ (co-culture of CsgA-α and CsgA-γ) and CsgA-$\alpha\gamma$-mix (1 h mixing of separately cultured CsgA-α and CsgA-γ). The viscosity of CsgA-$\alpha\gamma$-mix is similar to that of CsgA-$\alpha\gamma$.

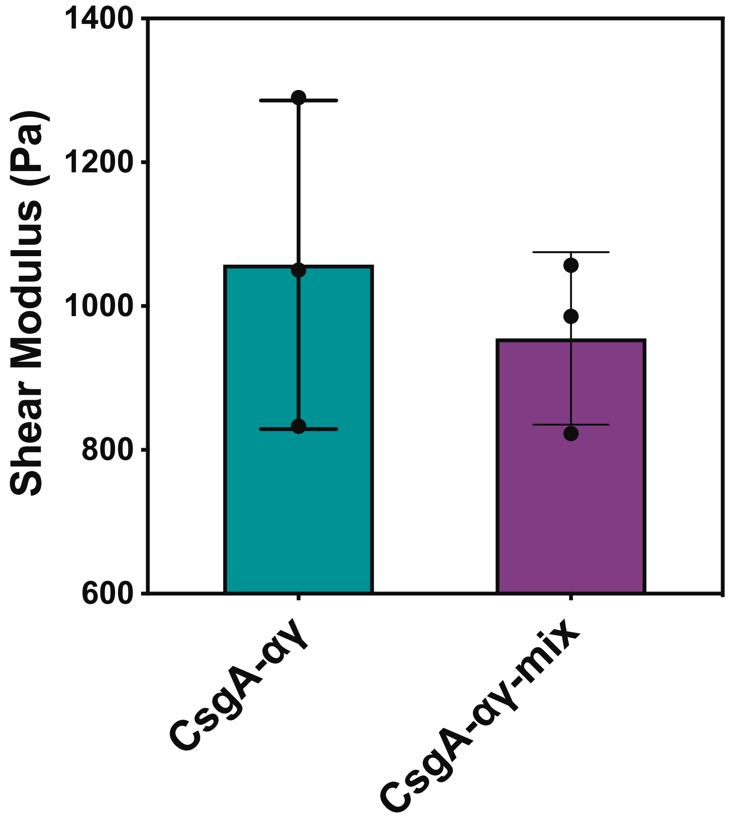

Supplementary Figure 3. Shear modulus of of CsgA-$\alpha\gamma$ and CsgA-$\alpha\gamma$-mix. The shear modulus of CsgA-$\alpha\gamma$-mix (1 h mixing of separately cultured CsgA-α and CsgA-γ) is similar to that of CsgA-$\alpha\gamma$ (co-culture of CsgA-α and CsgA-γ). n=3. Data represented as mean ± standard deviation.

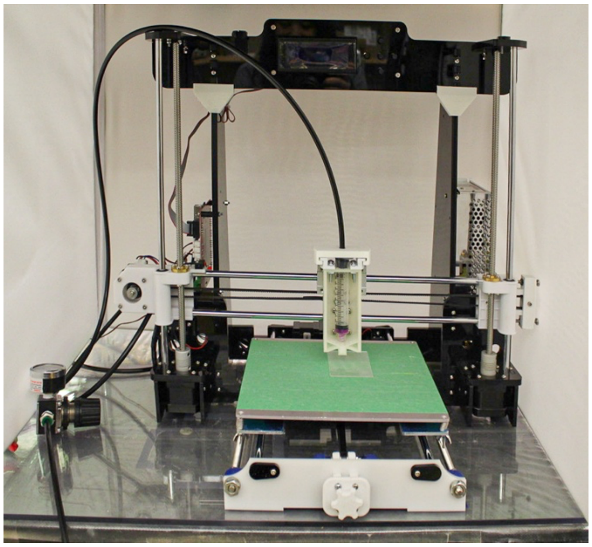

Supplementary Figure 4. Optical image of the customized 3D printer.

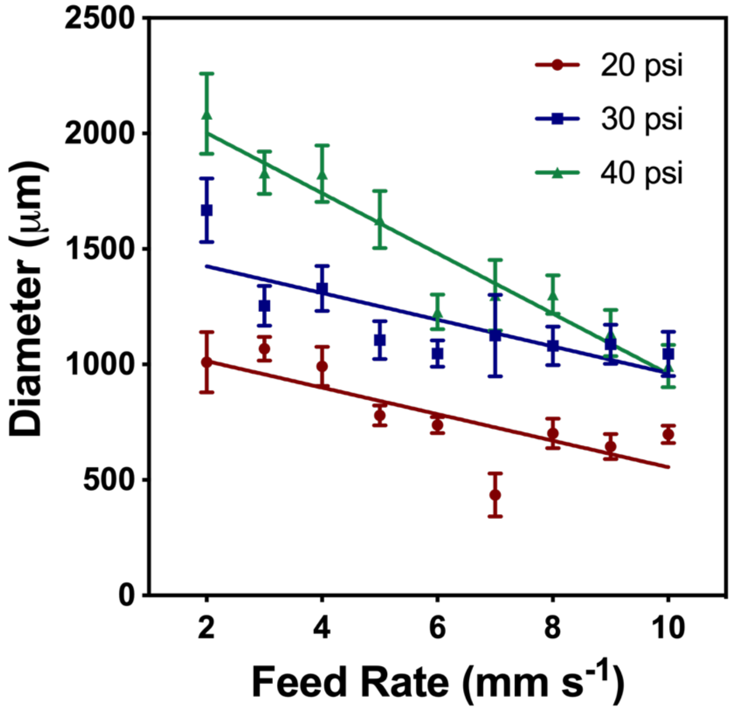

Supplementary Figure 5. Printing performance of CsgA-α. Plot shows the line width of CsgA-α hydrogel-based bioink at various feed rates (2-10 mm s^{-1}) and pressures (20-40 psi). n>10. Data represented as mean ± standard deviation.

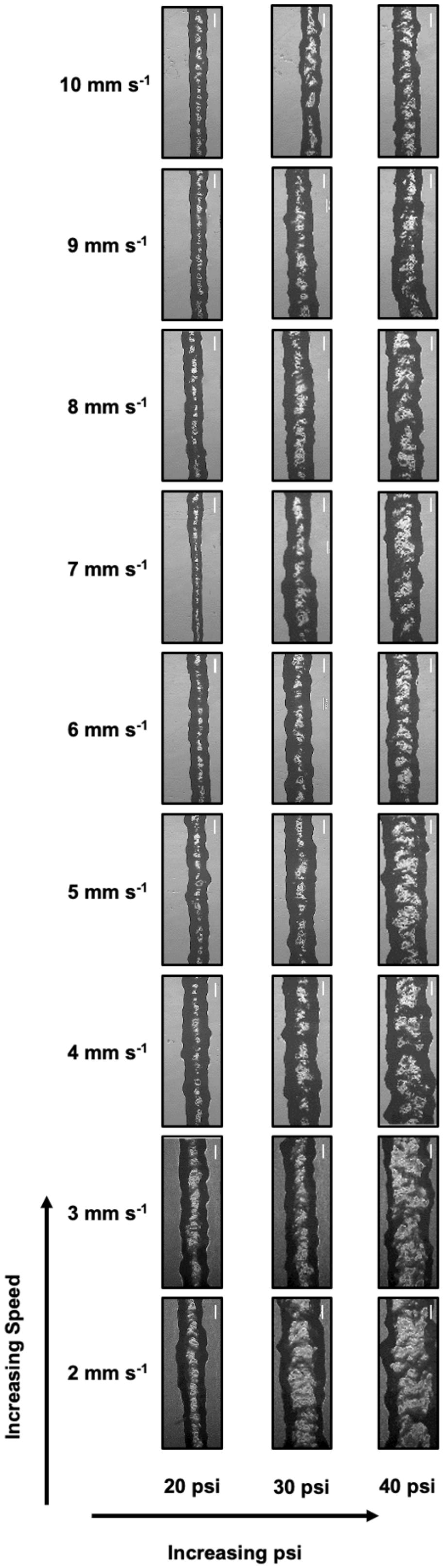

Supplementary Figure 6. Printing performance of CsgA-α. Optical images show the line width of CsgA-α hydrogel-based bioink at various feed rates (2-10 mm s^{-1}) and pressures (20-40 psi). Scale bar 500 μm.

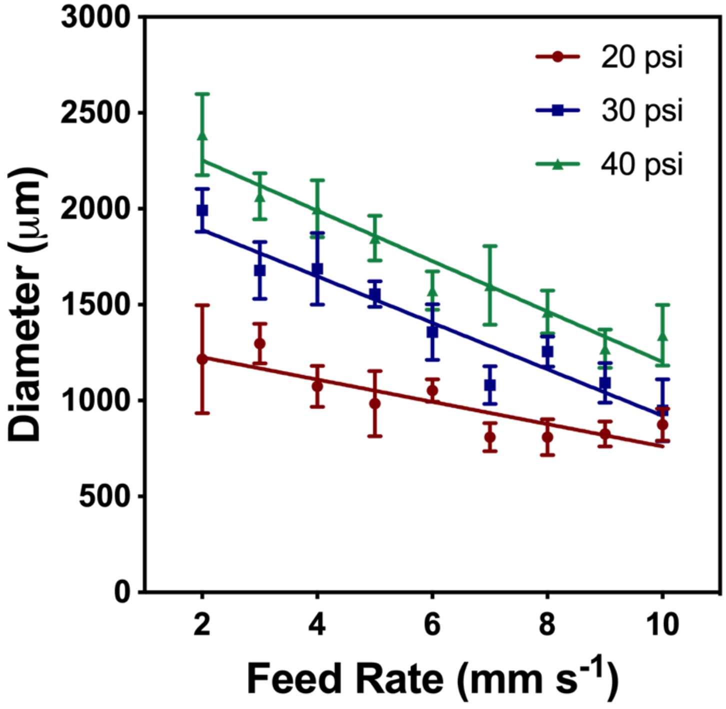

Supplementary Figure 7. Printing performance of CsgA-γ. Plot shows the line width of CsgA-γ hydrogel-based bioink at various feed rates (2-10 mm s^{-1}) and pressures (20-40 psi). n>10. Data represented as mean ± standard deviation.

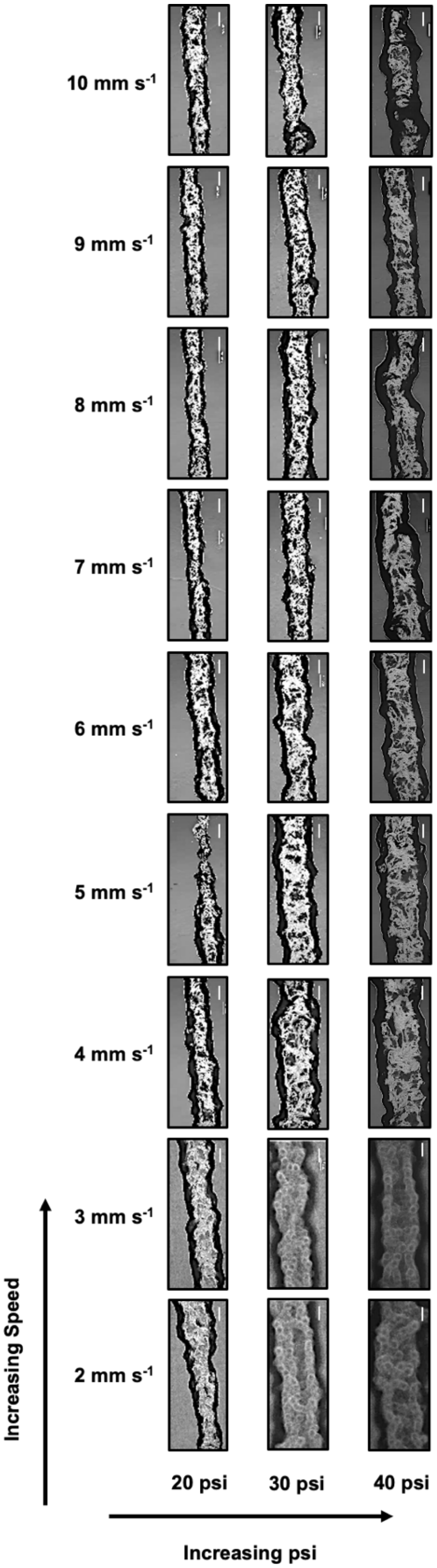

Supplementary Figure 8. Printing performance of CsgA-γ. Optical images show the line width of CsgA-γ hydrogel-based bioink at various feed rates (2-10 mm s^{-1}) and pressures (20-40 psi). Scale bar 500 μm.

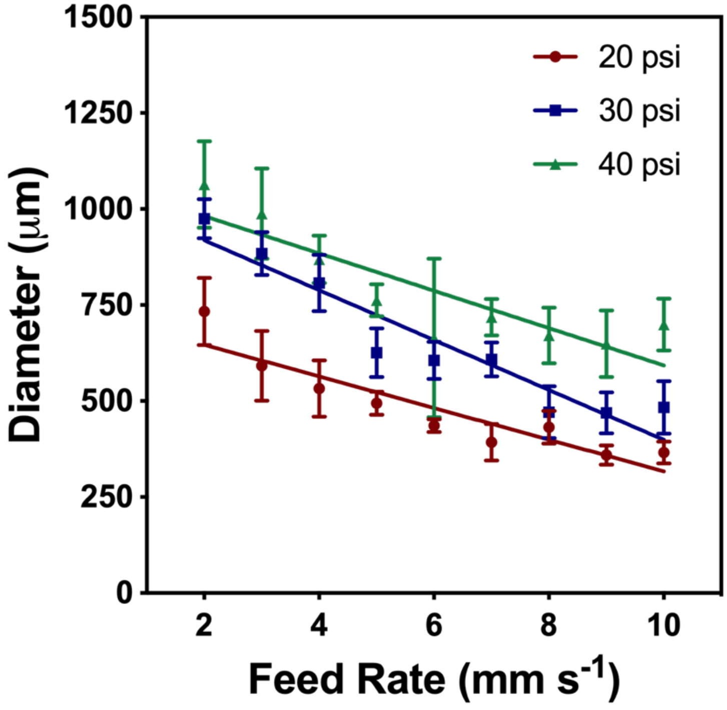

Supplementary Figure 9. Printing performance of CsgA-$\alpha\gamma$. Plot shows the line width of CsgA-$\alpha\gamma$ hydrogel-based bioink at various feed rates (2-10 mm s^{-1}) and pressures (20-40 psi). n>10. Data represented as mean ± standard deviation.

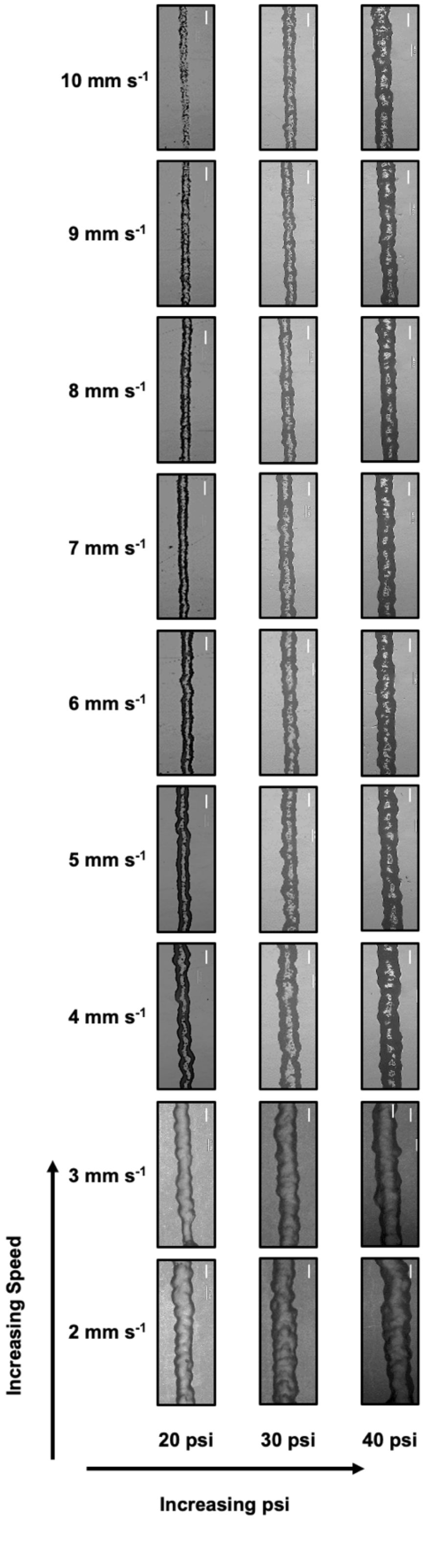

Supplementary Figure 10. Printing performance of microbial ink CsgA-$\alpha\gamma$. Optical images show the line width of CsgA-$\alpha\gamma$ bioink at various feed rates (2-10 mm s^{-1}) and pressures (20-40 psi). Scale bar 500 μm.

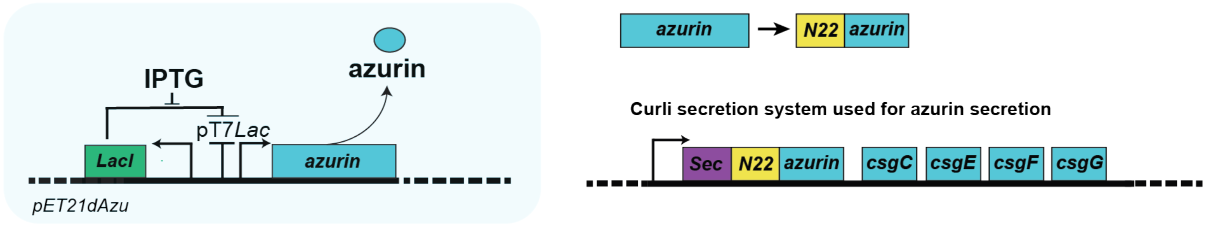

Name	Amino acid sequence
**Sec**	MKLLKVAAIAAIVFSGSALA
**N22**	GVVPQYGGGGNHGGGGNNSGPN
**Azurin[1]**	AECSVDIQGNDQMQFNTNAITVDKSCKQFTVNLSHPGNLPKNVMG HNWVLSTAADMQGVVTDGMASGLDKDYLKPDDSRVIAHTKLIGSG EKDSVTFDVSKLKEGEQYMSFCTFPGHSALMKGTLTLK

Supplementary Figure 11. Genetic design of pET21dAzu to secrete azurin. The anticancer biologic drug azurin was secreted via curli secretion system in PQN4-Azu cells by using pET21dAzu plasmid. The table shows the amino acid sequence of azurin.

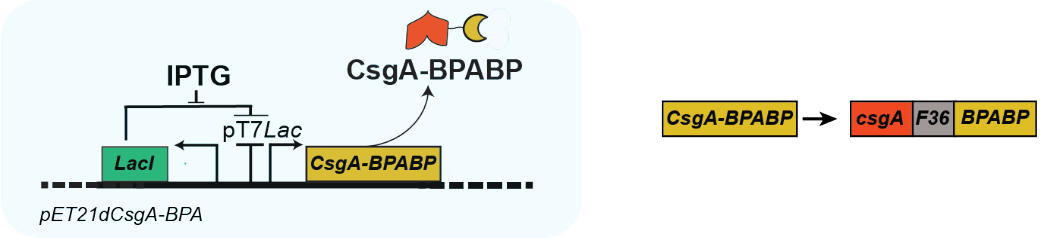

Name	Amino acid Sequence
**CsgA**	GVVPQYGGGGNHGGGGNNSGPNSELNIYQYGGGNSALALQTDAR<br>NSDLTITQHGGGNGADVGQGSDDSSIDLTQRGFGNSATLDQWNGK<br>NSEMTVKQFGGGNGAAVDQTASNSSVNVTQVGFGNNATAHQY
**Linker F36**	GGSGSSGSGGSGGGSGSSGSGGSGGGSGSSGSGGSG
**BPABP**[2]	KSLENSY

Supplementary Figure 12. Genetic design of pET21dCsgA-BPA to bind BPA. BPA binding peptide (BPABP) was genetically grafted to the CsgA protein to obtain PQN4-BPA biofilm. The table shows the amino acid sequence of CsgA-BPABP.

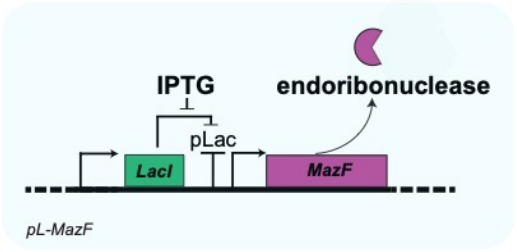

Name	Amino acid sequence
MazF	MVSRYVPDMGDLIWVDFDPTKGSEQAGHRPAVVLSPFMYNNKTG MCLCVPCTTQSKGYPFEVVLSGQERDGVALADQVKSIAWRARGAT KKGTVAPEELQLIKAKINVLIG

Supplementary Figure 13. Genetic design of pL-MazF to secrete MazF. The toxin MazF was expressed in PQN4-MazF cells by using pL-MazF plasmid. The table shows the amino acid sequence of MazF.

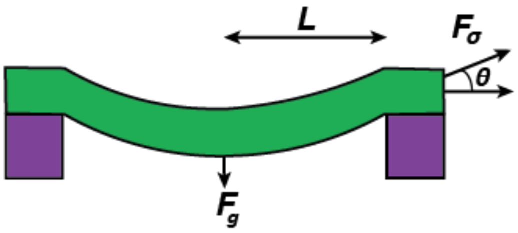

Supplementary Figure 14. Schematic of apparatus used for print test fidelity. The two vertical rectangles (purple) represent pillars across which the gel filament (green) has been extruded. The parameters used to create the plot in Figure 3h are indicated.

References

1 Yamada, T. *et al.* Apoptosis or growth arrest: Modulation of tumor suppressor p53's specificity by bacterial redox protein azurin. *Proc Natl Acad Sci U S A* **101**, 4770-4775, doi:10.1073/pnas.0400899101 (2004).

2 Maruthamuthu, M. K. *et al.* Development of bisphenol A-removing recombinant *Escherichia coli* by monomeric and dimeric surface display of bisphenol A-binding peptide. *Bioprocess Biosyst Eng* **41,** 479–487 (2018).
