# Supplementary material for: Programmable microbial ink for 3D printing of living materials produced from genetically engineered protein nanofibers
Source: Nat Commun. 2021 Nov 23;12:6600. doi: 10.1038/s41467-021-26791-x (PMC8611031; doi:10.1038/s41467-021-26791-x)
Supplement: Supplementary file 3 — Description of Additional Supplementary Files [file 41467_2021_26791_MOESM3_ESM.pdf]

Title: Supplementary Movie 1
Description: The video shows representative movies of the microbial ink fidelity test, wherein a filament composed of the CsgA-$\alpha\gamma$ ink is extruded as the printing nozzle moves (55 mm s^{-1}) across pillars with different spacings (largest gap = 16 mm). Subsequent video segments show examples of printing different two-dimensional and three-dimensional (i.e. multi-layered) structures.
