# Supplementary material for: Programmable microbial ink for 3D printing of living materials produced from genetically engineered protein nanofibers
Source: Nat Commun. 2021 Nov 23;12:6600. doi: 10.1038/s41467-021-26791-x (PMC8611031; doi:10.1038/s41467-021-26791-x)
Supplement: Supplementary file 4 — Reporting Summary [file 41467_2021_26791_MOESM4_ESM.pdf]

nature research

Corresponding author(s): Neel Joshi

Last updated by author(s): May 30, 2021

Reporting Summary

Nature Research wishes to improve the reproducibility of the work that we publish. This form provides structure for consistency and transparency in reporting. For further information on Nature Research policies, see our Editorial Policies and the Editorial Policy Checklist.

Statistics

For all statistical analyses, confirm that the following items are present in the figure legend, table legend, main text, or Methods section.

n/a	Confirmed	
☐	☒	The exact sample size (n) for each experimental group/condition, given as a discrete number and unit of measurement
☐	☒	A statement on whether measurements were taken from distinct samples or whether the same sample was measured repeatedly
☐	☒	The statistical test(s) used AND whether they are one- or two-sided<br>*Only common tests should be described solely by name; describe more complex techniques in the Methods section.*
☒	☐	A description of all covariates tested
☒	☐	A description of any assumptions or corrections, such as tests of normality and adjustment for multiple comparisons
☐	☒	A full description of the statistical parameters including central tendency (e.g. means) or other basic estimates (e.g. regression coefficient) AND variation (e.g. standard deviation) or associated estimates of uncertainty (e.g. confidence intervals)
☐	☒	For null hypothesis testing, the test statistic (e.g. F, t, r) with confidence intervals, effect sizes, degrees of freedom and P value noted<br>*Give P values as exact values whenever suitable.*
☒	☐	For Bayesian analysis, information on the choice of priors and Markov chain Monte Carlo settings
☒	☐	For hierarchical and complex designs, identification of the appropriate level for tests and full reporting of outcomes
☒	☐	Estimates of effect sizes (e.g. Cohen's d, Pearson's r), indicating how they were calculated

Our web collection on statistics for biologists contains articles on many of the points above.

Software and code

Policy information about availability of computer code

Data collection	no software was used for data collection
Data analysis	GraphPad PRISM 8, TRIOS software, Solid works 3D design software

For manuscripts utilizing custom algorithms or software that are central to the research but not yet described in published literature, software must be made available to editors and reviewers. We strongly encourage code deposition in a community repository (e.g. GitHub). See the Nature Research guidelines for submitting code & software for further information.

Data

Policy information about availability of data

All manuscripts must include a data availability statement. This statement should provide the following information, where applicable:

- Accession codes, unique identifiers, or web links for publicly available datasets
- A list of figures that have associated raw data
- A description of any restrictions on data availability

All relevant data supporting the findings of this study are available within the Article and its Supplementary Information or from the corresponding authors upon request. Source data are provided with this paper.

Field-specific reporting

Life sciences study design

All studies must disclose on these points even when the disclosure is negative.

Sample size	Sample sizes were chosen based on previous variance from similar experiments.
Data exclusions	No data was exluded
Replication	Experiments were replicated independently at least three times. All data from all replicates are included in the manuscript and they are reproducible.
Randomization	No sample randomization was necessary because all of the data is based on quantitative readouts that cannot be influenced by experimenter bias. All samples involved in comparisons were prepared and treated identically.
Blinding	No blinding was necessary because all of the data is based on quantitative readouts that cannot be influenced by experimenter bias. All samples involved in comparisons were prepared and treated identically.

Reporting for specific materials, systems and methods

We require information from authors about some types of materials, experimental systems and methods used in many studies. Here, indicate whether each material, system or method listed is relevant to your study. If you are not sure if a list item applies to your research, read the appropriate section before selecting a response.

Materials & experimental systems

n/a	Involved in the study	
☐	☒	Antibodies
☒	☐	Eukaryotic cell lines
☒	☐	Palaeontology and archaeology
☒	☐	Animals and other organisms
☒	☐	Human research participants
☒	☐	Clinical data
☒	☐	Dual use research of concern

Methods

n/a	Involved in the study	
☒	☐	ChIP-seq
☒	☐	Flow cytometry
☒	☐	MRI-based neuroimaging

Antibodies

Antibodies used	Goat Polyclonal Anti-Azurin Antibody (Origene AB0048-100) Dilution:1000. Rabbit anti-goat IgG (H+L) Secondary Antibody, HRP Dilution:1000 (Abcam ab6741)
Validation	This primary antibody was validated by the manufacturer against secreted azurin via Western Blot. This secondary antibody was validated by the manufacturer against polyclonal goat antibody (ab23938) via Western Blot.
