# Supplementary material for: Programmable microbial ink for 3D printing of living materials produced from genetically engineered protein nanofibers
Source: Nat Commun. 2021 Nov 23;12:6600. doi: 10.1038/s41467-021-26791-x (PMC8611031; doi:10.1038/s41467-021-26791-x)
Supplement: Supplementary file 5 — Source Data [file 41467_2021_26791_MOESM5_ESM.zip › 313106_2_related_ms_5981248_r15mkv.docx]

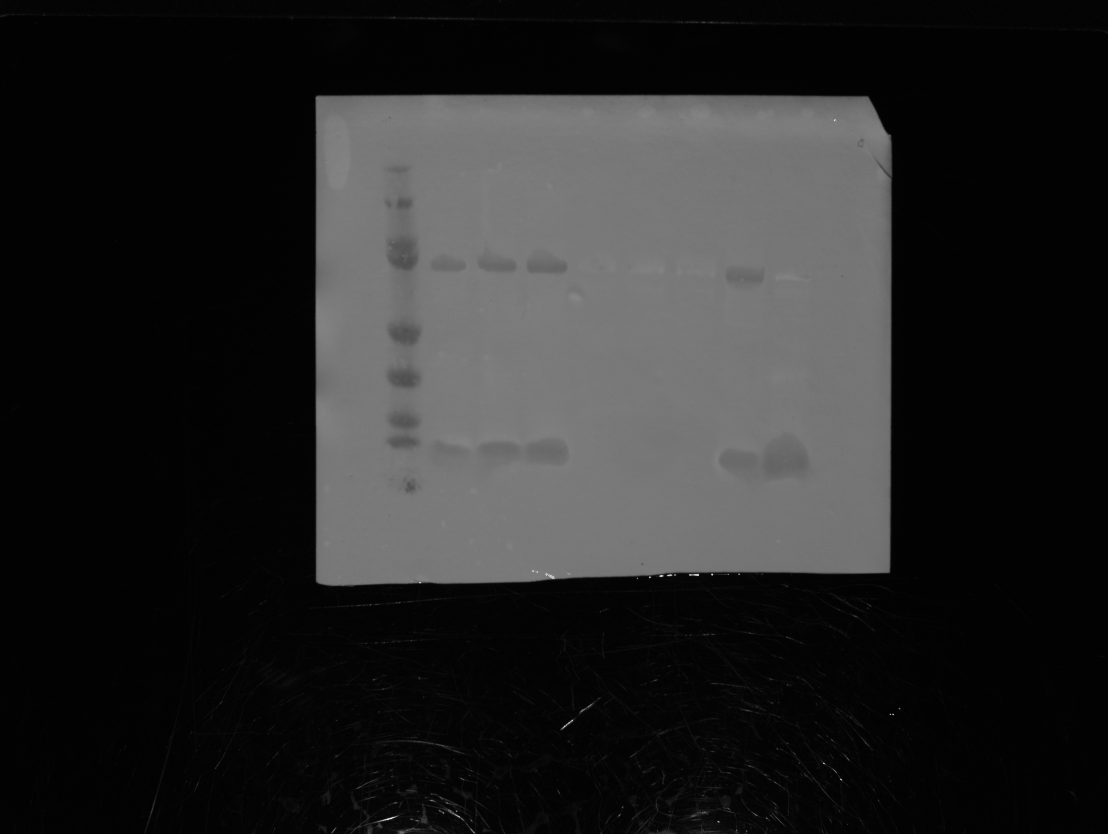


8

7

6

5

4a

3

2

1a

~ 120
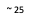

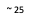


~ 85
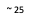

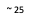


~ 60
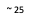

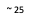


~ 50
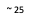

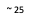


~ 40
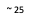

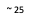


~ 25

~ 20

~ 15

~ 10

kDa

1-3 - azurin detected in the supernatant of the printed structure with IPTG induction

4-6 - azurin detected in the supernatant of the printed structure without IPTG induction

7 - azurin detected in the supernatant of the culture of bacteria with IPTG induction

8 - azurin detected in pellet of bacteria culture with IPTG induction
