# Supplementary figures and images for: Programmable microbial ink for 3D printing of living materials produced from genetically engineered protein nanofibers
Source: Nat Commun. 2021 Nov 23;12:6600. doi: 10.1038/s41467-021-26791-x (PMC8611031; doi:10.1038/s41467-021-26791-x)

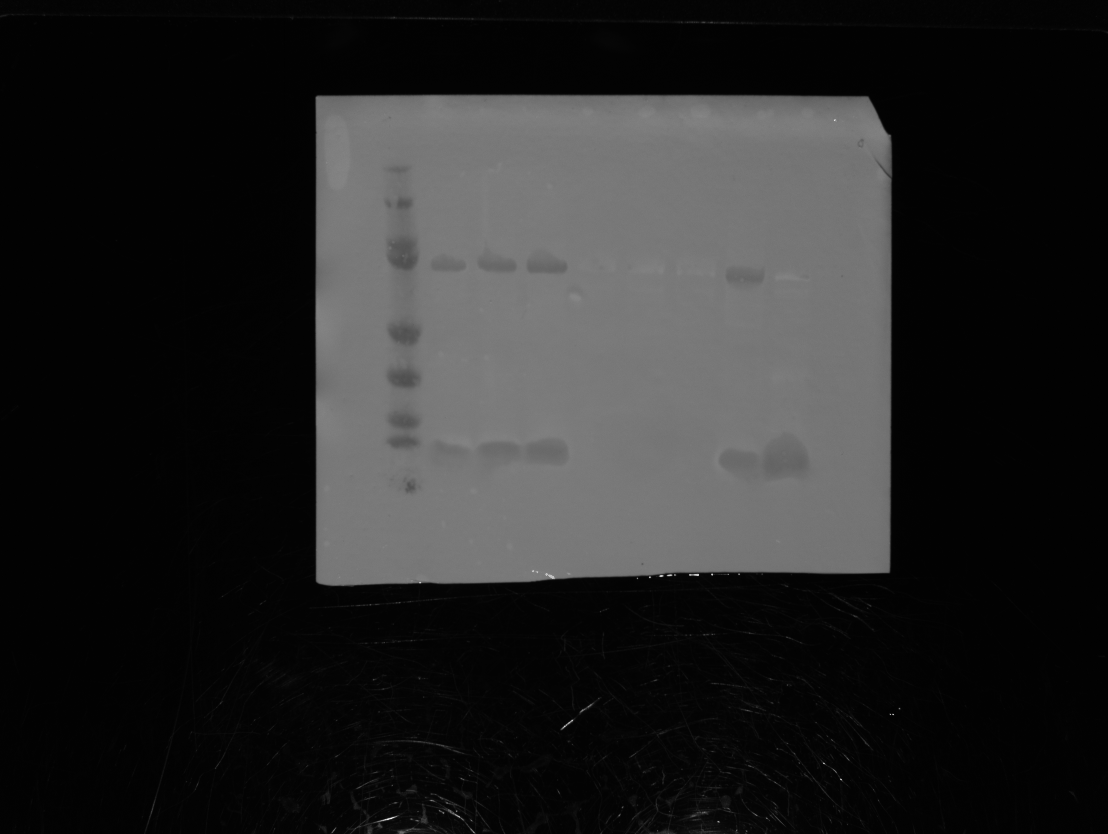

Supplement: Supplementary file 5 — Source Data [file 41467_2021_26791_MOESM5_ESM.zip › 313106_2_related_ms_5983264_r16jcf.png]
