# Supplementary material for: Programmable microbial ink for 3D printing of living materials produced from genetically engineered protein nanofibers
Source: Nat Commun. 2021 Nov 23;12:6600. doi: 10.1038/s41467-021-26791-x (PMC8611031; doi:10.1038/s41467-021-26791-x)
Supplement: Supplementary file 6 — Peer Review File [file 41467_2021_26791_MOESM6_ESM.pdf]

# Peer Review File

## Programmable Microbial Ink for 3D Printing of Living Materials Produced from Genetically Engineered Protein Nanofibers

**Open Access** This file is licensed under a Creative Commons Attribution 4.0 International License, which permits use, sharing, adaptation, distribution and reproduction in any medium or format, as long as you give appropriate credit to the original author(s) and the source, provide a link to the Creative Commons license, and indicate if changes were made. In the cases where the authors are anonymous, such as is the case for the reports of anonymous peer reviewers, author attribution should be to 'Anonymous Referee' followed by a clear attribution to the source work. The images or other third party material in this file are included in the article's Creative Commons license, unless indicated otherwise in a credit line to the material. If material is not included in the article's Creative Commons license and your intended use is not permitted by statutory regulation or exceeds the permitted use, you will need to obtain permission directly from the copyright holder. To view a copy of this license, visit http://creativecommons.org/licenses/by/4.0/.

Reviewers' Comments:

Reviewer #1:
Remarks to the Author:
In this paper, authors present a new system to produce engineered bacterial nanofibers compatible with 3D printers. These nanofibers are composed by genetically modified curly fibers able to cross-link between them. As a result, the authors can produce a new extracellular matrix with optimal properties in terms of viscoelastic performance. The cross-link strategy is based on the secretion of two different types of fibers composed by two different modified proteins, i.e. CsgA-α and CsgA-γ respectively. CsgA-α fibers can cross-link with CsgA-γ fibers. This cross-link occurs when both types of fibers are secreted in a co-culture or when are secreted separately and later mixed. Finally, different type of modified bacteria are successfully combined with the extracellular matrix.
There is no doubt about the interest of producing new biomaterials with new properties and functionalities. In this context, there are abundant literature related with the use of curly fibers for different purposes. However, it should be noted that the cross-link strategy presented is original. Despite this originality there are several aspects that must be addressed in this study.

1. Regarding the production and cross-link efficiency when both strains (secreting CsgA-α and CsgA-γ respectively) are co-cultured I wonder about the culture stability. In principle, the optimal performance will be obtained when the same amount of CsgA-α fibres and CsgA-γ fibers are produced. Have both strains the same growth rate and similar fibers secretion rate? How can you monitor this? When co-cultured, the ratio between both strains populations remains stable with time?
These questions are relevant in order to determine if co-culture of both type of fibers is better that independent culture and subsequent mixing. Which are the more efficient method?

2. Hydrogels production requires bacteria culture filtration to get higher nanofibers concentration. Which is the nanofibers production yield? Which is the relationship between culture volume and hydrogel production?

3. This study analyses several rheological features of the new biomaterial. However, the analysis performed, compare characteristic of the biomaterial composed by cross-linked curly fibers with respect the non-linked ones. In my opinion, this comparison is very limited and does not provide relevant information of the potential of this biomaterial. It should be much more interesting comparing the rheological properties of the biomaterial presented with other well-studied biomaterials such as, bacterial nanocellulose, collagen matrix, etc.

4. Related with the previous question, the analysis of other relevant properties such as, temporal and thermal stability of the printed structures, melting point, UV resistance, etc, would be of great interest.

5. I wonder about rigidity of the printed structures. Looking at the photos and video, they seem to be gelatinous. Are the printed structures rigid enough to be manipulated? How many drying time is necessary?

6. Regarding the combination of different engineered bacteria with the hydrogels, the advantages of these combinations are not clear for me. For instance, which are the advantages of producing azurin by using bacteria embedded in this matrix instead in a cell culture? The same question can be formulated with respect to the expression of MazF. Some potential examples of applications or advantages of using a combination of the engineered bacteria with the hydrogel in 3D printed structures should be described.

7. More interesting is the example of bacteria producing fibres displaying BPA-binding peptide (CsgA-BPABP). Can this material be used to produce membranes able to filter media removing BPA from it?

8. In table S1 there is a discrepancy between the order of the rows and the order of sequences represented in the figure. In the table, linker F12 is located after N22 but in the figure is located

before. Which is the right order?

Reviewer #2:
Remarks to the Author:
The authors present a quite novel approach to bio printing and bioink production. There have been multiple studies which show a range of techniques of producing "bioinks" but these are usually limited by the fact that they are usually polymer compounds encasing microbial cells. However, the authors of this paper present a truly engineered bioink, comprising of both ENGINEERED and FUNCTIONALISED microbes that produce their own bioink comprising of E.coli strains producing CsgA monomers which have been elegantly been fused to fibrin α-knob and γ-hole to generate a self-assembling, cross-linking and controllable biomaterial. Without the use of expensive industrial machinery and postproduction processing, as is the case with other biomaterials, to produce a bioink giving it a much more small-scale appeal which is useful for ease of production to allow studies of various biomaterial properties. They carry out extensive rheological analysis and then provide it with bioremediation, therapeutic and controllable functionality provided by the expression of heterologous proteins bringing about these functions.

The first experiments describe the production of this extrudable bioink formed by the non-covalent cross linking of polymerised secreted self-assembling CsgA-α and CsgA-γ fusion monomeric proteins, the α and γ domains derived from fibrin found in the extracellular matrix, which act like a "knob and hole" mechanism for non-covalent bonding. They found that strains expressing CsgA-α and CsgA-γ were less efficient at producing a stable biomaterial compared to a co-culture of these two strains, this adds to their conclusion that the fibrin domains are required for a stable biomaterial to be made. The microscopy images sufficiently prove that their co culture system works well to produce this biomaterial. They then go on to describe the extrusion and rheological characteristics of the material.

By studying the storage modulus, loss modules under frequency sweep and oscillatory sweep they are able to characterise the shear function and modulus of the bioink. Their data shows significant improvement in all analytical methods of rheology when comparing the CsgA-α and CsgA-γ to the co-culture CsgA-αγ bioink. They then further go on to test the filament collapse and the same pattern is observed adding to their hypothesis of fibrin domains increasing structural integrity of this extrudable bioink. The last experiment carried out in this portion shows a range of structures and layers made using the printing technique. These experiments prove a significant structural advantage and stability when using the co-cultured system. The final experiments focus on the functionalisation of the bioink.

Using the bioink they engineered a strain which expressed an azurin protein under the inducible control of the IPTG/pLac system. The Azurin protein is a well-known anticancer biologic drug. They showed successful secretion of Azurin via western blot once induced by IPTG. The second functionalisation they carried out was the use of bisphenol-A binding peptide fused to a CsgA monomer being secreted to produce the bioremediatory effect. Again, under the induction of IPTG the production of these fusion monomers provided a sequestration of biphenol-A compared to the non-fused microbial ink. Lastly, the control over population using the expression of endoribonuclease showed to have considerable impact on the CFUs once IPTG induced the expression of endoribonuclease. The authors demonstrated the ability to bioremediate, synthesis a therapeutic bioink and control the number of cells present within the bioink, all whilst extruding them into various printed images.

The aim of the paper as stated by the authors is to 1) produce a printable bioink with high fidelity. 2) produce a bioink ENTIRELY from engineered microbes using a bottom-up approach. 3) create a programmable platform that would enable novel functions for macroscopic 3D living architectures. All three of these aims fall into the category of novel aims and build upon the field of 3D bioprinting considerably, especially when thinking about "bioinks" as purely inks made of biological materials. The aims are fulfilled in the paper quite nicely and they have provided the first step in true programmable biomaterial production.

The readers of your journal will enjoy and appreciate a fully protein biomaterial produced by microbes themselves without the requirement for any chemical processing downstream of the production compared to biomaterials such as spider silk and "FLINK", a biomaterial which is largely based on chemical hydrogels with incorporated microbes. This is a important leap forward in the 3D printing field and fills a gap which has been present for a while. The functionalisation of this also yields space for adaption and improvement something readers will utilise and finally the clarity of the paper and quality of the figures allows for a pleasant reading experience. A simple and elegant paper which would be appreciated by readers of the journal.

Figure 1: Shows a brilliant and clear overview of experimental design, production, and functionalisation of the bioink. It clearly shows how the constructs are going to be designed and their overarching workflow as well as inspiration of the material. A very visually appealing and informative figure, complimented by references and explanation in the manuscript.

Figure 2: A nice microscopy image showing that the cross-linking behaviour that is desired can be inferred from these images. However, a critical point here is that the microscopy images do not necessarily mean that cross-linking is taking place. However, the results do support their hypothesis.

Figure 3: A thorough analysis of the rheological characteristics of the single CsgA-$\alpha$ and CsgA-$\gamma$ variants. This coincides with the work from Figure 2, providing more evidence that their "CsgA-knob-hole" crosslinking is taking place and influencing the rheological characteristics of the bioink.

Figure 4: A brilliant set of experiments showing significant integration and production of azurin, sequestration of bisphenol A compared to the stand alone bioink, and finally a significant control overgrowth of E. coli within the bioink.

Suggested improvements to be made:

1. Line 282: "co-culture of CsgA-$\alpha$ and CsgA-$\gamma$"- the culture is defined as a "co-culture". Is there an auxotrophic or other dependency between the strains which allows for control over populations of the two cultures or are we seeing an uncontrolled mixed culture?

2. Figure 2: Shows the conjugation of the CsgA-$\alpha\gamma$ microbial ink but the measure to this is using a microscopy image? Is there no other method to prove that conjugation is providing this increase in nm width that is being observed, such as an SDS page gel with CsgA monomers with the hole and knobs being shown to conjugate and seeing this size difference on a gel?

3. Figure 4: A slight clarification as to why the growth of E.coli is useful in this context, does it provide an advantage for structural control over the bioink's rheological properties?

4. The conclusion is very nicely written and the majority of it is agreed upon, however there is an over estimation of real-world applications, specifically its use in terrestrial and extra-terrestrial building. What is the cost of the production of this bioink? How can it be used to make structures when it's only been shown to work at a small scale? Why would this be chosen over other 3D printable non-biomaterials and what benefit would this give to a structure being a biomaterial? And compared to industrial standards what is the benefit of it? We agree that this currently has very good applicability to bioremediation and therapeutics, however the use in structural building is a bit reaching, at least with the current data present.

5. In the experiments which show functionalisation, the strains used to produce the bioink are not used to carry out the functions and instead a separate culture is used and mixed with the bioink producing PQN4 E. coli strains. Can the authors elucidate as to why they weren't engineered using the CsgA-$\alpha$ or CsgA-$\gamma$ secreting strains? It would seem a more elegant design if they were all produced using the same strain producing the bioink, this indicates an issue when tried? What was this issue?

**Programmable Microbial Ink for 3D Printing of Living Materials**
**Produced from Genetically Engineered Protein Nanofibers**

**Reviewer #1 (Remarks to the Author):**

In this paper, authors present a new system to produce engineered bacterial nanofibers compatible with 3D printers. These nanofibers are composed by genetically modified curly fibers able to cross-link between them. As a result, the authors can produce a new extracellular matrix with optimal properties in terms of viscoelastic performance. The cross-link strategy is based on the secretion of two different types of fibers composed by two different modified proteins, i.e. CsgA-α and CsgA-γ respectively. CsgA-α fibers can cross-link with CsgA-γ fibers. This cross-link occurs when both types of fibers are secreted in a co-culture or when are secreted separately and later mixed. Finally, different type of modified bacteria are successfully combined with the extracellular matrix.
There is no doubt about the interest of producing new biomaterials with new properties and functionalities. In this context, there are abundant literature related with the use of curly fibers for different purposes. However, it should be noted that the cross-link strategy presented is original. Despite this originality there are several aspects that must be addressed in this study.

**We appreciate the remarks of Reviewer #1 and the following responses addresses all the queries of the Reviewer.**

1. Regarding the production and cross-link efficiency when both strains (secreting CsgA-α and CsgA-γ respectively) are co-cultured I wonder about the culture stability. In principle, the optimal performance will be obtained when the same amount of CsgA-α fibres and CsgA-γ fibers are produced. Have both strains the same growth rate and similar fibers secretion rate? How can you monitor this? When co-cultured, the ratio between both strains populations remains stable with time?
These questions are relevant in order to determine if co-culture of both type of fibers is better that independent culture and subsequent mixing. Which are the more efficient method?

**CsgA-α and CsgA-γ are the constructs that produce engineered curli fibers from the same *E. coli* strain namely PQN4, and moreover, these two cultures exhibited similar bacterial growth and expression kinetics. The 48 h cultures of both CsgA-α and CsgA-γ resulted in similar wet biomass of ~2.5 g from 500 ml cultures. Moreover, the congo red assay of 500 ml 48 h cultures of CsgA-α and CsgA-γ were also similar, wherein the binding of congo red dye to the CsgA (amyloid) is utilized as a measure of the production of the engineered curli fibers. However, it should be noted that it is not feasible to monitor the production of CsgA-α and CsgA-γ independently in the co-culture using congo red assay, as the dye is specific to amyloid and not to α or γ protein fusions.**

**The rheological studies presented in Figures 3a-h and the Supplementary Figures 2&3 show that the co-culture CsgA-αγ is similar if not slightly better compared to subsequently mixed culture CsgA-αγ-mix. These results indicate the specific interactions of α and γ modules to cross link effectively in 1 h mixing of separately cultured CsgA-α and CsgA-γ. Furthermore, we do not think it is likely nor is it essential to have a 1:1 ration of alpha and gamma variants in order to affect mechanical properties significantly.**

2. Hydrogels production requires bacteria culture filtration to get higher nanofibers concentration. Which is the nanofibers production yield? Which is the relationship between culture volume and hydrogel production?

**The production of hydrogels from the bacterial cultures using the filtration system has been reported by us previously (Advanced Materials 2019, 31, 1901826). By using our congo red assay, we estimate that the curli nanofibers account for ~20% of the wet biomass of the culture. However, it should be noted that the E. coli cells, and the nanofibers get filtered out to different extents during the production of the hydrogel by our filtration protocol. We are able to produce 1-2 ml of hydrogel from 1 L of bacterial culture with a gelation concentration of 3-5%.**

3. This study analyses several rheological features of the new biomaterial. However, the analysis performed, compare characteristic of the biomaterial composed by cross-linked curly fibers with respect the non-linked ones. In my opinion, this comparison is very limited and does not provide relevant information of the potential of this biomaterial. It should be much more interesting comparing the rheological properties of the biomaterial presented with other well-studied biomaterials such as, bacterial nanocellulose, collagen matrix, etc.

**In this study, we have reported the microbial ink that is developed entirely from genetically engineered microbial cells. As our microbial ink was the first of its kind, we have presented the detailed rheological characterization and its printability. As the focus of our paper was on bioinks comprising of microbial cells, in the introduction, we have given exhaustive descriptions about the comparable inks and their performance. These examples of the bioinks not only addresses the Reviewer #1's remarks on comparing the microbial ink with other bioinks that are made of nanocellulose, alginate, hyaluronic acid *etc.*, but also involves examples of different 3D printing techniques and strategies for the ink formulations. In addition, we have given references (ref # 1,2 & 11) to bioink reviews that provide additional examples of bioinks for tissue engineering and other biomedical applications. It should also be noted that it is difficult to make a direct comparison with other bioinks/biomaterials due to their differences in the formulations and the printing settings.**

4. Related with the previous question, the analysis of other relevant properties such as, temporal and thermal stability of the printed structures, melting point, UV resistance, etc, would be of great interest.

**The primary focus of this work was to produce an extrudable bioink directly from the engineered bacteria and to facilitate 3D printing of functional living architectures. As our microbial ink was intended for printing living microbial cells-which are in general sensitive to temperature and UV radiation, we did not explore the thermal stability and UV resistance of our microbial ink, in this current study. However, we would like to mention that the curli fibers are known to be highly robust due to their resistance to heat, solvents, denaturants, pH and detergents. Our group and others have explored these robust characteristics of engineered curli fibers in our previous publications (Adv Mater, 2019, 31, 1901826; Nat Chem Biol, 2021, 17, 732). We have added a sentence in the introduction to emphasize this point:**

***"It should also be noted that fibers formed from the self-assembly of CsgA are highly stable and resistant to proteolytic, detergent-induced, and heat-based denaturation.*[30]**"**

5. I wonder about rigidity of the printed structures. Looking at the photos and video, they seem to be gelatinous. Are the printed structures rigid enough to be manipulated? How many drying time is necessary?

**As shown in Figures 3i-l, the printed structures were rigid enough to retain their 3D shape and structure, that included a 21-layered solid cone and 10-layered circle/square. In addition, the fidelity test presented in Figure 3g and the Supplementary Video further demonstrated the rigidity of the microbial ink to self-stand instantaneously and no drying time was necessary. However, as the microbial ink comprises of ~ 95% water, the printed structures will dry out eventually, even while retaining their rough shape and form.**

6. Regarding the combination of different engineered bacteria with the hydrogels, the advantages of these combinations are not clear for me. For instance, which are the advantages of producing azurin by using bacteria embedded in this matrix instead in a cell culture? The same question can be formulated with respect to the expression of MazF. Some potential examples of applications or advantages of using a combination of the engineered bacteria with the hydrogel in 3D printed structures should be described.

**We take the reviewer's point that the specific demonstrations we include with respect to embedded cells do not all have clear applications. Nevertheless, the reviewer themselves identified the potential utility of one of our demonstrations – BPA binding – in that it could be used to produce membranes or other devices for BPA removal in different contexts. The other two demonstrations – azurin production and MazF-induced cell death – are included more to demonstrate the ability of embedded cells within the structure to: 1) remain viable; 2) secrete proteins into the surrounding space; and, 3) limit their own survivability outside the printed structure. We think these demonstrations, while not immediately applicable to any mature technology, are central to the core concepts of Engineered Living Materials, namely building structures that leverage the responsive potential of living cells within them. The demonstrations may also be useful for downstream applications like biocontainment, co-culturing different cell types (e.g. bacteria and mammalian) while preventing physical contact, and environmentally responsive biomedical devices for implantation.**

7. More interesting is the example of bacteria producing fibres displaying BPA-binding peptide (CsgA-BPABP). Can this material be used to produce membranes able to filter media removing BPA from it?

**Yes. We envision that this sequestration living material can be developed as 3D-printed water filters to remove various contaminants including BPA.**

8. In table S1 there is a discrepancy between the order of the rows and the order of sequences represented in the figure. In the table, linker F12 is located after N22 but in the figure is located

before. Which is the right order?

**We thank the reviewer for pointing out this discrepancy. We have now corrected the order of sequences in the table.**

**Reviewer #2 (Remarks to the Author):**

The authors present a quite novel approach to bio printing and bioink production. There have been multiple studies which show a range of techniques of producing “bioinks” but these are usually limited by the fact that they are usually polymer compounds encasing microbial cells. However, the authors of this paper present a truly engineered bioink, comprising of both ENGINEERED and FUNCTIONALISED microbes that produce their own bioink comprising of E.coli strains producing CsgA monomers which have been elegantly been fused to fibrin $\alpha$-knob and $\gamma$-hole to generate a self-assembling, cross-linking and controllable biomaterial. Without the use of expensive industrial machinery and postproduction processing, as is the case with other biomaterials, to produce a bioink giving it a much more small-scale appeal which is useful for ease of production to allow studies of various biomaterial properties. They carry out extensive rheological analysis and then provide it with
bioremediation, therapeutic and controllable functionality provided by the expression of heterologous proteins bringing about these functions.

The first experiments describe the production of this extrudable bioink formed by the non-covalent cross linking of polymerised secreted self-assembling CsgA-$\alpha$ and CsgA-$\gamma$ fusion monomeric proteins, the $\alpha$ and $\gamma$ domains derived from fibrin found in the extracellular matrix, which act like a “knob and hole” mechanism for non-covalent bonding. They found that strains expressing CsgA-$\alpha$ and CsgA-$\gamma$ were less efficient at producing a stable biomaterial compared to a co-culture of these two strains, this adds to their conclusion that the fibrin domains are required for a stable biomaterial to be made. The microscopy images sufficiently prove that their co culture system works well to produce this biomaterial. They then go on to describe the extrusion and rheological characteristics of the material.

By studying the storage modulus, loss modules under frequency sweep and oscillatory sweep they are able to characterise the shear function and modulus of the bioink. Their data shows significant improvement in all analytical methods of rheology when comparing the CsgA-$\alpha$ and CsgA-$\gamma$ to the co-culture CsgA-$\alpha\gamma$ bioink. They then further go on to test the filament collapse and the same pattern is observed adding to their hypothesis of fibrin domains increasing structural integrity of this extrudable bioink. The last experiment carried out in this portion shows a range of structures and layers made using the printing technique. These experiments prove a significant structural advantage and stability when using the co-cultured system. The final experiments focus on the functionalisation of the bioink.

Using the bioink they engineered a strain which expressed an azurin protein under the inducible control of the IPTG/pLac system. The Azurin protein is a well-known anticancer biologic drug. They showed successful secretion of Azurin via western blot once induced by IPTG. The second functionalisation they carried out was the use of bisphenol-A binding peptide fused to a CsgA monomer being secreted to produce the bioremediatory effect. Again, under the induction of IPTG the production of these fusion monomers provided a sequestration of biphenol-A compared to the non-fused microbial ink. Lastly, the control over population using the expression of endoribonuclease showed to have considerable impact on the CFUs once IPTG

induced the expression of endoribonuclease. The authors demonstrated the ability to bioremediate, synthesis a therapeutic bioink and control the number of cells present within the bioink, all whilst extruding them into various printed images.

The aim of the paper as stated by the authors is to 1) produce a printable bioink with high fidelity. 2) produce a bioink ENTIRELY from engineered microbes using a bottom-up approach. 3) create a programmable platform that would enable novel functions for macroscopic 3D living architectures. All three of these aims fall into the category of novel aims and build upon the field of 3D bioprinting considerably, especially when thinking about “bioinks” as purely inks made of biological materials. The aims are fulfilled in the paper quite nicely and they have provided the first step in true programmable biomaterial production.

The readers of your journal will enjoy and appreciate a fully protein biomaterial produced by microbes themselves without the requirement for any chemical processing downstream of the production compared to biomaterials such as spider silk and “FLINK”, a biomaterial which is largely based on chemical hydrogels with incorporated microbes. This is a important leap forward in the 3D printing field and fills a gap which has been present for a while. The functionalisation of this also yields space for adaption and improvement something readers will utilise and finally the clarity of the paper and quality of the figures allows for a pleasant reading experience. A simple and elegant paper which would be appreciated by readers of the journal.

**We are overwhelmed and greatly appreciate the detailed remarks of reviewer #2 about our work, which will further motivate us to design and develop novel living materials in the future!**

Figure 1: Shows a brilliant and clear overview of experimental design, production, and functionalization of the bioink. It clearly shows how the constructs are going to be designed and their overarching workflow as well as inspiration of the material. A very visually appealing and informative figure, complimented by references and explanation in the manuscript.

**We thank reviewer #2 for this comment.**

Figure 2: A nice microscopy image showing that the cross-linking behaviour that is desired can be inferred from these images. However, a critical point here is that the microscopy images do not necessarily mean that cross-linking is taking place. However, the results do support their hypothesis.

**We agree with the reviewer #2 that the microscopy images do not necessarily confirm the crosslinking, which we have proved by the rheological studies and fidelity tests.**

Figure 3: A thorough analysis of the rheological characteristics of the single CsgA-$\alpha$ and CsgA-$\gamma$ variants. This coincides with the work from Figure 2, providing more evidence that their “CsgA-knob-hole” crosslinking is taking place and influencing the rheological characteristics of the bioink.

**We thank the reviewer #2 for this comment.**

Figure 4: A brilliant set of experiments showing significant integration and production of azurin, sequestration of bisphenol A compared to the stand alone bioink, and finally a significant control overgrowth of E. coli within the bioink.

**We appreciate reviewer #2 for this comment.**

Suggested improvements to be made:

1. Line 282: "co-culture of CsgA-α and CsgA-γ"- the culture is defined as a "co-culture". Is there an auxotrophic or other dependency between the strains which allows for control over populations of the two cultures or are we seeing an uncontrolled mixed culture?

**The co-culture of CsgA-α and CsgA-γ were uncontrolled mixed cultures. We didn't control the co-culture of CsgA-α and CsgA-γ, as the engineered fibers are produced from the same *E .coli* strain PQN4 and moreover, the bacterial growth and expression kinetics of these two cultures are very similar. The rheological characteristics of microbial ink CsgA-αγ are due to the supramolecular crosslinking (knob-hole interactions) of the alpha and gamma domains, which was also reflected in CsgA-αγ-mix that was obtained by mixing separate cultures of CsgA-α and CsgA-γ. Thus, we felt that regulation of co-cultures CsgA-α and CsgA-γ was not necessary for this initial demonstration. Further work that controls the ratio of bacterial types with auxotrophy or some other mechanism could offer the ability to tune materials properties.**

2. Figure 2: Shows the conjugation of the CsgA-αγ microbial ink but the measure to this is using a microscopy image? Is there no other method to prove that conjugation is providing this increase in nm width that is being observed, such as an SDS page gel with CsgA monomers with the hole and knobs being shown to conjugate and seeing this size difference on a gel?

**The curli fibers are resistant to SDS and to disassemble CsgA variants so that they migrate into the SDS-PAGE gel, curli fibers need to be incubated in the hexafluoroisopropanol (HFIP) solvent and formic acid (FA) to release monomeric CsgA. This treatment of curli fibers in the harsh condition facilitates disassembly of fibers, but also disrupts the knob-hole interaction and therefore validation of alpha-gamma crosslinking by SDS-PAGE is not feasible. On the other hand, other techniques like FTIR, Raman etc. are not useful to detect the changes in signals arising from supramolecular crosslinking of alpha-gamma interactions in the backdrop of other protein-protein and molecular interactions. In the context of our manuscript we think the electron microscopy coupled with the clear trends in rheology – regardless of whether the bacteria are co-cultured or mixed after culturing – are sufficient data to support our conclusion.**

3. Figure 4: A slight clarification as to why the growth of E.coli is useful in this context, does it provide an advantage for structural control over the bioink's rheological properties?

**The presence of engineered *E. coli* in the microbial ink do not provide structural control on its own but can facilitate the production or templating of structural components. However, the regulation of growth of *E. coli* in the microbial ink was demonstrated as an**

**additional function. This regulation becomes important to tailor the microbe (*E. coli*) numbers within the living architecture *e.g.* bioproduction (as described in ref 3), while some biomedical applications might require killing of all the living cells after achieving a desired function.**

4. The conclusion is very nicely written and the majority of it is agreed upon, however there is an over estimation of real-world applications, specifically its use in terrestrial and extra-terrestrial building. What is the cost of the production of this bioink? How can it be used to make structures when it's only been shown to work at a small scale? Why would this be chosen over other 3D printable non-biomaterials and what benefit would this give to a structure being a biomaterial? And compared to industrial standards what is the benefit of it? We agree that this currently has very good applicability to bioremediation and therapeutics, however the use in structural building is a bit reaching, at least with the current data present.

**We agree with Reviewer #2 that in this work, we have not demonstrated the applications of our microbial ink for terrestrial and extra-terrestrial building. However, in an on-going work from our lab, we have utilized the microbial ink to 3D print strong solid composites for structural material applications, which will be published separately. In this work, we have only suggested that microbial ink could be used as a "promising platform" for developing microbial manufacturing technology for structural materials. There is also considerable interest in the literature, wherein few groups (Ref #36) are also using bacteria for such structural applications.**

**Regarding the cost, we would like to mention that the presented microbial ink was developed in a laboratory setting, while the use of bioreactors could significantly increase the yield and reduce the cost of microbial ink. The microbial ink or any other engineered living material provides several advantages over other biomaterials or non-biomaterials through their living characteristics such as self-replication, self-regulation, self-healing, environment-responsiveness and sustainable production. Further, any extra-terrestrial building applications can not be accomplished by transporting all the raw materials from earth and therefore the engineered microbes that can self-regenerate and make use of the raw materials available in space for structural applications appears to be the most promising avenue.**

**To address the reviewer's comment, we have added a qualifying statement to the relevant conclusion statement:**
**"*Especially if combined with other materials technologies, such as those that are already incorporating living cells into structural building materials, our microbial bioink could also be particularly useful for structure building in space or extraterrestrial habitats, where raw material transport is extremely difficult, making on-demand generation of building materials from very limited resources essential.*"**

5. In the experiments which show functionalisation, the strains used to produce the bioink are not used to carry out the functions and instead a separate culture is used and mixed with the bioink producing PQN4 E. coli strains. Can the authors elucidate as to why they weren't engineered using the CsgA-$\alpha$ or CsgA-$\gamma$ secreting strains? It would seem a more elegant design

if they were all produced using the same strain producing the bioink, this indicates an issue when tried? What was this issue?

**We agree with Reviewer #2 that one can make strains that can serve multiple functions such as producing the microbial ink and any other targeted functions, as described in Figure 4. However, in this work, our primary goal was to demonstrate the production of the microbial ink entirely from the engineered microbes. Secondly, we wanted to showcase a plug-and-play design, wherein the microbial ink can be embedded with any engineered microbes to obtain the desired functions independently. On the other hand, engineered microbes having multiple functions might require additional strain engineering to optimize the production and/or secretion. Moreover, the production of other functional components (*e.g.* azurin) along with engineered curli nanofibers might become unnecessary, for example during the production of microbial ink by filtration, wherein the cells are lysed and in turn limits the usage of living cells that can be regulated using appropriate inducers.**

Reviewers' Comments:

Reviewer #1:
Remarks to the Author:
The authors have properly addressed all the comments raised by the reviewer.

Reviewer #2:
Remarks to the Author:
The authors have done a good job explaining their replies to my concerns and updating the manuscript. Congratulations to the team for the great work on this nice paper.
